# Development of hybrid biomicroparticles: cellulose exposing functionalized fusion proteins

**DOI:** 10.1186/s12934-024-02344-x

**Published:** 2024-03-14

**Authors:** Joanna Żebrowska, Piotr Mucha, Maciej Prusinowski, Daria Krefft, Agnieszka Żylicz-Stachula, Milena Deptuła, Aneta Skoniecka, Agata Tymińska, Małgorzata Zawrzykraj, Jacek Zieliński, Michał Pikuła, Piotr M. Skowron

**Affiliations:** 1 Department of Molecular Biotechnology, Faculty of Chemistry, University of Gdansk, Gdansk, 80-308 Poland; 2 BioVentures Institute Ltd, Poznan, 60-141 Poland; 3 Department of Molecular Biochemistry, Faculty of Chemistry, University of Gdansk, Gdansk, 80-308 Poland; 4 Laboratory of Tissue Engineering and Regenerative Medicine, Division of Embryology, Faculty of Medicine, Medical University of Gdansk, Gdansk, 80-211 Poland; 5 Division of Clinical Anatomy, Faculty of Medicine, Medical University of Gdansk, Gdansk, 80-211 Poland; 6 Department of Oncologic Surgery, Faculty of Medicine, Medical University of Gdansk, Gdansk, 80-211 Poland

**Keywords:** Cellulose biomicroparticles, Cellulose-binding-domain fusions, DNA-FACE technology, Concatemeric proteins

## Abstract

**Background:**

One of the leading current trends in technology is the miniaturization of devices to the microscale and nanoscale. The highly advanced approaches are based on biological systems, subjected to bioengineering using chemical, enzymatic and recombinant methods. Here we have utilised the biological affinity towards cellulose of the cellulose binding domain (CBD) fused with recombinant proteins.

**Results:**

Here we focused on fusions with ‘artificial’, concatemeric proteins with preprogrammed functions, constructed using DNA FACE™ technology. Such CBD fusions can be efficiently attached to micro-/nanocellulose to form functional, hybrid bionanoparticles. Microcellulose (MCC) particles were generated by a novel approach to enzymatic hydrolysis using *Aspergillus sp.* cellulase. The interaction between the constructs components – MCC, CBD and fused concatemeric proteins – was evaluated. Obtaining of hybrid biomicroparticles of a natural cellulose biocarrier with proteins with therapeutic properties, fused with CBD, was confirmed. Further, biological tests on the hybrid bioMCC particles confirmed the lack of their cytotoxicity on 46BR.1 N fibroblasts and human adipose derived stem cells (ASCs). The XTT analysis showed a slight inhibition of the proliferation of 46BR.1 N fibroblasts and ACSs cells stimulated with the hybrid biomicroparticles. However, in both cases no changes in the morphology of the examined cells after incubation with the hybrid biomicroparticles’ MCC were detected.

**Conclusions:**

Microcellulose display with recombinant proteins involves utilizing cellulose, a natural polymer found in plants, as a platform for presenting or displaying proteins. This approach harnesses the structural properties of cellulose to express or exhibit various recombinant proteins on its surface. It offers a novel method for protein expression, presentation, or immobilization, enabling various applications in biotechnology, biomedicine, and other fields. Microcellulose shows promise in biomedical fields for wound healing materials, drug delivery systems, tissue engineering scaffolds, and as a component in bio-sensors due to its biocompatibility and structural properties.

**Supplementary Information:**

The online version contains supplementary material available at 10.1186/s12934-024-02344-x.

## Background

### Bionanoparticles – functional molecules

This work is a continuation of research concerning the construction of concatemeric proteins (DNA FACE™ technology) previously published in the Materials Science and Engineering C [1]. Currently, nanomaterials composed of nanoparticles are one of the fastest-growing and most intensively studied groups of materials [2, 3]. Nanomaterials’ unique physicochemical and biological features such as their tunable size, charge and shape, surface structure, chemical composition, solubility in biological fluids, biocompatibility, non-toxicity and ability to interact with biomolecules, have made them widely used in many fields of modern science, including biomedicine [4–6]. Advances in nanotechnology are aiming at a combination of scaling down to a sub-100 nm size while incorporating novel complex features, not found in original materials used for nanoparticles’ construction. Thus far, an astonishing amount of nanomaterials, ranging from inorganic nanoparticles to polymeric biological structures and synthetic nanostructured ultra-thin films with various electronic, magnetic, optical and (bio-) chemical properties are being developed. These developments have found an application not only for next-generation nanoelectronic devices, nanomaterials generally, but also in pharmaceuticals including next-generation drug delivery systems, regenerative medicine, cosmetics, food biotechnology and diagnostics. Particularly interesting is the possibility of building molecules with new multifunctional properties on one surface side, providing a matrix for embedded or surface immobilised (bio-) nanoparticles. Despite the fact that various materials (metals, silica, synthetic polymers) were used to design and synthesise nanoparticles, it seems that bionanoparticles containing biomolecules naturally occurring in the cell, such as lipids, peptides, proteins or polysaccharides, are characterised by excellent biological properties [7–9]. For this reason, they are the most used and promising tools in biomedicine. However, with the advent of genetic engineering, phage display systems and evolution in vitro, recombinant, modified constructs seem an alternative, the importance of which will rapidly increase. The degree of complexity of the structures formed by bionanoparticles is extremely wide, from relatively simple structures such as micelles or liposomes, to much more complex ones, such as fibrils or virus-like particles [10–12]. Such structures are the subject of intensive research aimed at testing their applicability as new drug delivery systems to specific locations in a cell or organism [13, 14].

### Cellulose as a new biomaterial of biomedical applications and a main component of controllable drug delivery systems

The increasing demand for biocompatible materials capable of replacing plastics is gaining more attention for natural polymers such as cellulose [15–18]. Cellulose is the most abundant biopolymer on the Earth [19, 20]. It originates mostly from wood and plants and some species of bacteria where it is synthesised as an extracellular biopolymer that performs various biological functions [21]. Cellulose is built of a long unbranched chain consisting of repeating anhydroglucose structural units linked by a β-1,4-glycosidic bond. Unmodified cellulose has a limited use [22, 23]. However, its tunable properties may be modified in terms of physical or chemical properties [15, 16, 24–27]. Such modified cellulose-based biomaterials offer large advantages over known conventional ones and show great promise in medical applications. Due to cellulose’s biodegradable and non-toxicity properties, its biocompatibility and its hydrophilicity, it has been widely used as dressings for difficult-to-heal wounds, tissue engineering scaffolds or controllable drug delivery systems [16, 28, 29]. Reducing the size of cellulose particles is one of the ways of modifying their physicochemical parameters. The micro- and nanometer-sized cellulose particles form a scaffold of many materials for biomedical applications [30–33]. One of the methods of their design and preparation (apart from chemical hydrolysis) is the enzymatic hydrolysis of cellulose with the use of bacterial or fungal cellulases, enzymes that hydrolyze β-type glycosidic bonds, shortening the length of the chain and the cellulose particle dimension. As a result of enzymatic hydrolysis, micro- or nanocellulose is obtained with properties desirable in biomedical applications [34–37]. The biocompatible, biodegradable, and bioavailable features of modified cellulose-based materials make it a promising proposal to design a new type of a controlled drug delivery system [16, 28, 38]. The tunable properties of cellulose and its ability for chemical modifications allow it to tune such critical parameters like the place, dose, time and velocity of drug release. The stability of a pharmacologically active substance may be also controlled [38, 39]. For this reason, cellulose is of great interest as a remarkable natural biopolymer that can selectively and in a controlled manner deliver drugs to the desired location in the body or cell.

### Characterization of the cellulose binding domain (CBD)

A typical cellulolytic enzyme consists of a distinct CBD and at least one of several distinct catalytic domains that are structurally and functionally separate units of proteins and are linked by a CBD interdomain peptide linker [40]. Only cellulases from a few microorganisms and higher plants do not have such domains [41]. According to the amino acid sequence similarity and the 3D structure of the adsorption module, different types of cellulases from different sources have been classified into families [42].

In the latest update of the CAZY CBM database, they are grouped into 100 families (18.12.2023 r.) that show significant differences in their substrate specificity and exhibit different biological functions. Data and classification with regard to this can be found on the server of the carbohydrate binding module family (http://www.cazy.org/CBM1_all.html). The affinity and specificity for cellulose may differ [43]. However, it goes on to state that the specificity of CBD (cellulose-binding domain) can be altered or modified through genetic engineering by introducing simple mutations [44]. Essentially, it suggests that while the natural affinity and specificity for cellulose may vary, it is possible to customize or adjust the specificity of CBD for cellulose through genetic engineering techniques involving minor genetic changes. CBDs from different families are structurally similar, and their ability to bind to cellulose depends on several aromatic amino acids [43]. Thus, their specificity can be modified by mutagenesis and exchange of amino acids responsible for the ability to bind to cellulose [44]. The affinity of CBD proteins for cellulose can depend on several factors: the structure of the CBD, the linker between the CBD and the target protein, the pH of the solution, ionic strength, temperature, and the ratio of protein-CBD concentration to cellulose [43].

Currently, there are over 200 putative sequences identified in over 40 different species. The binding domains are classified into different families based on the amino acid sequence, binding specificity, and structure [43]. Families V and VIII consist of only one member, while Families I, II and III consist of 40 or more members [45]. The relative molecular weight of CBD ranges from 0.4 × 10^4^ to 2.0 × 10^4^ Da [43]. CBD can contain 30–180 amino acids present as a single, double, or triple domain in a single protein. Their localization in the parent protein can be C- or N-terminal and sometimes centrally in the polypeptide chain. Most CBDs have a β-sandwich structure and vary in the number of residues, while some families have a β-trefoil, a unique cysteine knot, a β-barrel and lectin-like folds [43, 46, 47].

The bond between CBD and cellulose is formed through a combination of hydrophobic and polar interactions. Specifically, Van der Waals interactions are emphasized as playing a crucial role in stabilizing the CBD-cellulose bond. Additionally, it mentions that CBD and cellulose fibers can create a series of hydrogen bonds due to electrostatic interactions [40, 43]. In summary, the bonding between CBD and cellulose involves a complex interplay of hydrophobic forces, polar interactions, Van der Waals forces, and hydrogen bonds, contributing to the overall stability of the bond [48–50].

**Fig. 1:**
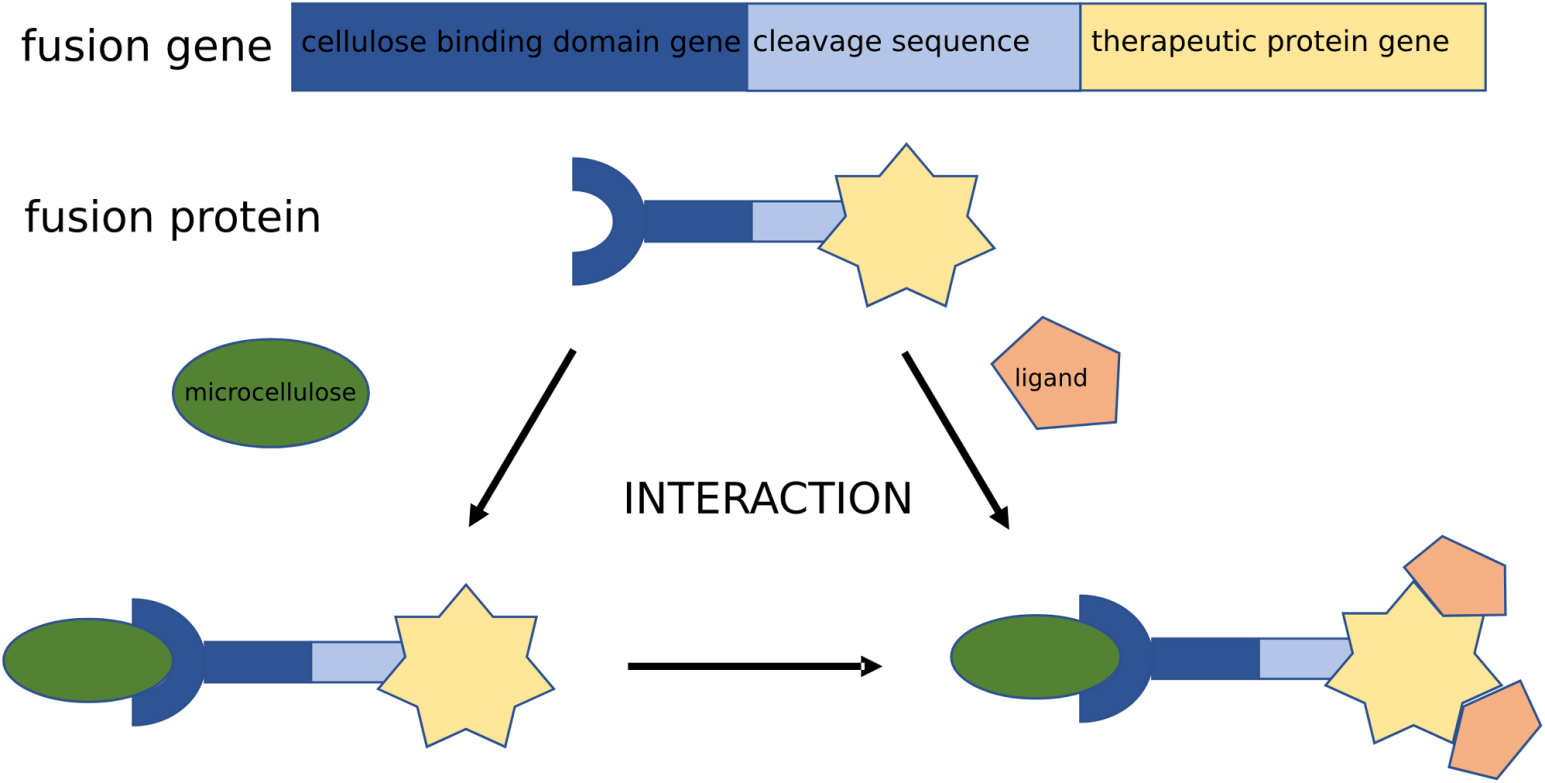
Schematic representation of the construction of a hybrid bioMCC (or bionanocellulose). The drawing shows an interaction of a CBD-fusion protein with a biocompatible MCC (nanocellulose) carrier. This construct can be subjected to further enhancement by the binding of a functionalized ligand (e.g. therapeutic), which exhibits an affinity toward the CBD-fusion protein

A CBD was first identified in the fungus *Trichoderma reesei* [51] and the bacterium *Cellulomonas fimi* [52]. The existence of a linker between the CBD moiety and the catalytic part of the cellulase was then proved. Forty years after proposing the cellulase molecule model, it was cloned from *Clostridium cellulovorans* and *C. fimi* [53]. CBD was found in hydrolytic and non-hydrolytic proteins. In proteins with hydrolytic activity (cellulase and xylanase), CBD is the domain that concentrates catalytic domains on the surface of an insoluble cellulosic substrate. In proteins that do not have hydrolytic activity, CBD is part of a scaffold subunit, organizing the catalytic subunits into a coherent complex of multienzymes. CBD has been identified in hemicellulase, endomannanase and acetyloxylanesterase. CBD has also been recognized in xylanase derived from *Clostridium thermocellum* [54], esterases from *Penicillium funiculosum* and pectinate lyase in *Pseudomonas cellulose* [55]. Moreover, there is the presence of such domains in the b-glucosidase located in *Phanerochaete chrysosporium* [56]. The presence of putative CBD in plant endoglucanases has also been reported. Expansins, which are believed to play a role in the non-hydrolytic expansion of the cell wall, are CBD homologs and have the ability to bind cellulose [43]. In recombinant fusion proteins, the CBD domain may be placed at the N- or C-terminus of the protein, preferably separated by a linker or protease recognition sequence (Fig. 1). The most common method of purifying such fusion proteins is cellulosic affinity chromatography through the formed interactions between CBD and cellulose chains [43].

### Polyepitopic, concatameric proteins with therapeutic properties

In this study, a unique DNA FACE™ technology developed in our laboratory [1, 57–59] for the construction of concatemeric proteins was used. This is an *Escherichia coli*-based DNA amplification-expression method allowing for the automatic assembly of concatemeric Open Reading Frames (ORFs) and proteins, based on the intertwined use of specialised vectors, containing universal DNA amplification modules, transcription/translation modules and two enzymes: SapI Type IIS restriction endonuclease, and T4 DNA ligase. The module contains two convergent DNA recognition sequences for SapI, separated by a SmaI site for the insertion of any DNA fragment and a cassette site of the gene into a vector. For the purpose of this work, the vectors were modified to include a DNA segment encoding the CBD domain to create fusion proteins that interact with cellulose nanoparticles. The DNA FACE™ method has many potential applications in: (i) pharmaceutical and tissue engineering, (ii) vaccine production, (iii) drug delivery systems, (iv) toxins and metal ions removal from human’s, animal’s bodies and the environment and (iv) the mass production of peptide biomaterials. The technology is protected by RP, USA, EU, Indian, Japanese, Israeli patents and Chinese patent application [60–62].

In our study, we worked with concatemeric proteins composed of multiplied epitopes with general regenerative or neuroregenerative properties are derived from the human angiopoietin like 6 protein (ANGPTL6) fragment 337–346 (labelled as AGF) and the arginine-glycine-aspartic acid peptide motif (RGD) [63, 64]. Such functionalized CBD fusions exhibit the ability to bind various cellulose forms, including their microparticles.

## Materials and methods

### Bacterial strains, cell lines, plasmid, media, reagents, procedures

#### Biochemical reagent

The materials were composed of: *Aspergillus sp*. cellulase (Merck, Darmstadt, Germany, cat. no. C2605), commercial MCC preparations (SigmaCell 20, Merck, cat. no. S3504), human serum sample (Merck, Darmstadt, Germany, cat. no. H4522), mineral oil (Merck, Darmstadt, Germany, cat. no. M5904), silica capillary (Postnova Analytics GmbH, Germany). The capillary electrophoresis (CE) analysis was performed on a P/ACE MDQ + System (Sciex, USA), controlled by Karat software. A Beckman Coulter LS13320 laser diffraction particle size analyser was used. The particle size analyser operates on the principle of laser radiation diffraction in accordance with ISO 13320:2009.

#### Biotechnological/biological reagents

All REases were from New England Biolabs (Ipswich, MA, USA). Q5 polymerase, T4 DNA Ligase, Shrimp Alkaline Phosphatase (SAP) were from New England Biolabs (Ipswich, MA, USA). 100 bp and 1 kb DNA and protein ladders were from Thermo Fisher Scientific Baltics UAB (Vilnus, Lithuania) and GE Healthcare (Upsalla, Sweden). Chromatographic media and columns: Ni HisTrap HP, HiLoad 16/600 Superdex 200 pg were from GE Healthcare (Upsalla, Sweden), Affi-Prep Polymyxin Resin Bio-Rad (Hercules, CA, USA). The 1 x PBS buffer of standard composition: 137 mM NaCl, 2.7 mM KCl, 10 mM Na_2_HPO_4_, and 1.8 mM KH_2_PO_4_, pH 7.4. High performance NGC Chromatography System from BioRad (Hercules, CA, USA). Vivaflow 50 Laboratory Cross Flow Cassette [PES] and Vivaspin® Turbo 15 [PES] centrifugal concentrators from Sartorius (Göttingen, Germany). NEB 5-alpha *E. coli* (derivative of DH5α) [*fhuA2 (argF-lacZ)U169 phoA glnV44 80 (lacZ)*M15 *gyrA96 recA1 relA1 endA1 thi-1 hsdR17*] from New England Biolabs (Ipswich, MA, USA) was used for plasmid DNA purification. *E. coli* strains (Invitrogen™ BL21 (DE3), Invitrogen™ BL21 Star (DE3), Agilent Technologies BL21-Gold (DE3) and ClearColi® BL21(DE3), F– *ompT hsdSB (rB- mB-) gal dcm lon* λ(DE3 [*lacI lac*UV5-T7 gene 1 *ind1 sam7 nin5*]) *msbA148* Δ*gutQ*Δ*kdsD* Δ*lpxL*Δ*lpxM*Δ*pagP*Δ*lpxP*Δ*eptA]* Lucigen) were used for gene expression. Horseradish peroxidase, anti-polyHistidine antibodies, Sigma FAST™ Protease Inhibitor, were from Sigma-Aldrich (St. Louis, MO, USA). Bacterial media components were from BTL (Lodz, Poland). UltraPure Agarose and UltraPure Low Melting Point Agarose were from Thermo Fisher Scientific (Waltham, MA, USA). DNA purification kits were from A&A Biotechnology (Gdansk, Poland). DNA sequencing and the oligos chemical synthesis was performed at Eurofins (Warsaw, Poland) or Eurofins Genomics (Ebersberg, Germany). Trans-Blot Turbo Mini PVDF Transfer Pack was from Bio-Rad (Hercules, CA, USA). The gene synthesis was performed at Gene Universal and Genescript (Piscataway NJ, USA). Other reagents were from Avantor Performance Materials Poland S.A. (Gliwice, Poland), AppliChem Inc. (St. Louis Missouri, MO, USA) or Fluka Chemie GmbH (Buchs, Switzerland). The genetic maps of the DNA vectors and recombinant constructs were performed using SnapGene software version 4.1 (https://www.snapgene.com). STATISTICA software was from StatSoft (Cracow, Poland). Prism 5 software was from GraphPad Software (San Diego, CA, USA). The 46BR.1 N cell line was purchased from The European Collection of Authenticated Cell Cultures (ECACC). Primaria flasks (cat. no 353,810) were from Becton Dickinson (Franklin Lakes, NJ, USA). Fetal bovine serum (FBS) for human cell culture, DMEM HG (Dulbecco’s Modified Eagle Medium High Glucose), cat. no D6429, 10% Fetal Bovine Serum, cat. no F9665, Cell Proliferation Kit II, cat no 11,465,015,001, and Histopaque® were from Sigma-Aldrich (St. Louis, MO, USA). Cell cytotoxicity test based on lactate dehydrogenase (LDH), cat. no MK401 was from Takara (Japan). Data were acquired using CytoFLEX S Flow Cytometer (Beckman Coulter) and analysed by analysis software (Kaluza). Cells were stained by Calcein AM (Thermo Fisher Scientific, Waltham, MA, USA, C3099) as a measure of enzymatic activity in live cells, and DAPI (Beckman Coulter, B30437) as a measure of dead cells with disturbed membrane integrity.

## Methods

### Capillary electrophoresis analysis of CBD_AGF_30_His and CBD_RGD_20_His proteins stability in PBS buffer and human serum

Analysis of CBD_AGF_30_His and CBD_RGD_20_His proteins stability in 0.1 × PBS buffer and human serum was performed using the CE method. The method of obtaining these proteins is described in later subsections. The serum was stored at -20 °C before use. The sample was then thawed at room temperature and centrifuged in a microcentrifuge before the experiment. Proteins after freezing in liquid nitrogen were stored at the same conditions and then an identical procedure was followed as for the serum sample.

In the initial CE experiment, the stability of the proteins was characterised in 0.1 × PBS pH 7.2. The analysis conditions were the same as those described below for studying the stability of these proteins in human plasma. To characterise protein stability in human serum 10 µl of CBD_AGF_30_His and CBD_RGD_20_His proteins (the concentration of each protein was 1 mg/ml) was mixed with a 10 µl of the plasma sample (1:1 *v/v* ratio) at room temperature. Subsequently, 5 µl of light mineral oil was placed on the sample surface to eliminate the evaporation effect of the sample at elevated temperature. Mixing was performed directly in the nanoVial, which was directly placed in the thermostated sample chamber of the P/ACE MDQ + Sciex capillary electrophoresis apparatus at 37 °C. Time-dependent protein stability analyses were performed on the same originally prepared sample. An uncoated, fused silica capillary, 50 cm (40 cm to detector) × 75 μm, stabilised at 25 °C, was used. Separations were performed at 20 kV with normal electrode polarisation (cathode at the detector end). 50 mM phosphate + 25 mM SDS, (pH 7.0) was used as a background electrolyte. Unmixed CBD_AGF_30_His and CBD_RGD_20_His proteins and plasma samples were also analysed under the same conditions. Samples were injected into the capillary at its anodic end *via* a hydrodynamic injection at 0.5 psi for 4 s. The capillary was rinsed with background electrolyte solution for 2 min between runs. The separation process was monitored with a UV detector at 214 nm. The automatic peaks integration method implemented in the Karat software was used to characterise the protein stability.

### Cellulose particle size analyses

Cellulose particle size analyses were performed using a laser size particle analyser type LS13320 Beckman-Coulter. Dynamic light scattering (DLS) experiments were performed at room temperature. Cellulose microparticles measurements were made on the Beckman Coulter LS13320 laser diffraction particle size analyser provided with an automatic module for wet powder. The particle size analyser operates on the principle of laser radiation diffraction in accordance with ISO 13320:2009. Performance of the analyser is verified against a certified standard every 2–3 months or whenever irregularities are observed, according to the test standard from Beckman Coulter.

### Preparation of fragmented MCC particles

*Aspergillus sp*. cellulase has been used for the controlled, partial degradation of commercial MCC preparations to obtain low diameter MCC particles. 1 ml of *Aspergillus sp.* cellulase enzyme preparation (≥ 1000 units) was added to 30 ml of 0.1 × PBS buffer (adjusted to pH 6.5 with phosphoric acid) containing 1 g dispersed cellulose (Sigmacell, type 20). The mixture was incubated in a Falcon probe at 30 °C for 3 weeks, while constantly stirring on a magnetic stirrer. All the biological experiments further described here concern fragmented MCC obtained in this work. The prepared MCC was radiation sterilised with a dose of 28 kGy at the Radiation Sterilisation Station for Medical Devices and Transplants (Warsaw, Poland).

### Preparation of pET28_delSapI_CBD_His and pET28_delSapI_MalE_CBD_His expression vectors

Two DNA cassettes containing: (i) the ORF encoding CBD from *Clostridium cellulovorans* followed by enterokinase site, and (ii) MalE leader followed by gene encoding CBD from *Clostridium cellulovorans* and enterokinase site, were designed and synthesized. The 5’ and 3’ ends of both cassettes contained the BsaI and XhoI restriction sites, respectively. DNA containing both cassettes were digested with BsaI and XhoI REases and gel purified. The pET28_delSapI expression vector [58] was digested with NcoI and XhoI REases, dephosphorylated with SAP and gel purified. The resultant DNA fragments were ligated with T4 DNA ligase. The ligated DNAs were transformed into *E. coli* NEB DH5-alpha chemicompetent cells. The obtained bacterial clones were used for plasmid DNA propagation and purification. The purified plasmid DNAs were screened by restriction mapping and DNA sequencing, yielding correct pET28_delSapI_CBD_His and pET28_delSapI_MalE_CBD_His expression vectors.

### Preparation of pET28_delSapI_MalE_CBD_AGF_X_His and pET28_delSapI_MalE_CBD_RGD_X_His vectors

The pET28_delSapI_MalE_CBD_His vector was SapI linearized, dephosphorylated with SAP and gel purified. The previously constructed plasmids pET28AMP_MalE_AGF_X and pET28AMP_PhoA_RGD_X [1, 58] were used to obtain the concatemeric DNA segments for further cloning. The selected plasmids contained 10, 20, 30 and 40 directed repeats of the sequences coding the RGD and TSRGDHELLGGAAPVGG peptides. Plasmids were SapI digested and DNA fragments were gel purified. The prepared inserts were ligated with the pET28_delSapI-MalE-CBD vector and the ligation mixtures were used for *E. coli* NEB DH5-alpha chemicompetent cells transformation.

### General scheme for directional DNA fragment amplification

The steps of the target gene amplification reaction include: (i) designing a DNA fragment (monomer) for amplification; (ii) chemical DNA synthesis, PCR amplification, or cloned monomer excision using restriction endonuclease; (iii) enriching the monomer with 3-nt asymmetric cohesive ends at the 5’ and 3’ ends, generated by SapI restriction endonuclease; (iv) purifying the DNA monomer equipped with 3-nt cohesive ends; (v) directional self-ligating of DNA monomers in a head-to-tail orientation; (vi) ligation of the mixture of the formed concatemers to an amplification-expression vector; (vii) selecting bacterial clones containing a concatemer with the desired monomer copy number; (viii) direct biosynthesis of the concatemeric protein encoded by the obtained DNA concatemer using strong vector promoters; (iv) - (viii), can be repeated until the desired number of monomer copies within the concatemer is obtained [58].

### Expression of gene coding recombinant concatemeric proteins: CBD_AGF_X_His and CBD_RGD_X_His and their isolation from periplasmic space

Biosynthesis of the fusion concatemeric proteins variants with 10, 20, 30 and 40 repeats of the sequences: RGD or AGF was performed at 37 °C in an LB medium, supplemented with kanamycin. The recombinant bacteria were cultivated until the A_600nm_ reached ca. 0.6. Then, the recombinant gene expression was induced with 1 mM IPTG. After induction, the bacterial cultures were continued for 2 h only at 30 °C as this is sufficient for MalE leader directing secretion of the concatemeric proteins into the periplasmic space. This was immediately followed by recombinant concatemeric proteins release from the periplasmic space using osmotic shock. 2 L of the culture was centrifuged (3500 × g, 10 min at 10 °C). The bacterial pellet was resuspended in 200 ml of a saccharose buffer at 20 °C, supplemented with a EDTA [20% saccharose, 20 mM Tris-HCl, pH 8.0 at 20 °C, 5 mM EDTA] and incubated at 20 °C for 10 min. The suspension was centrifuged (5000 × g, 25 min at 10 °C), then the bacterial pellet was resuspended in a 120 ml cold 10 mM MgCl_2_ buffer and incubated on ice for 10 min. The suspension was centrifuged (5000 × g, 10 min at 4 °C). The cell debris was removed by filtration through a 0.2 μm PES (polyethersulfone) membrane and the supernatant containing concatemeric proteins was subjected to purification.

### Protein purification and endotoxin removal

Concatemeric proteins: CBD_AGF_X_His and CBD_RGD_X_His isolated from the periplasmic space were further purified using a high performance NGC Chromatography System. A single purification step on immobilized metal ion affinity chromatography (IMAC) was sufficient for obtaining homogeneous protein. The osmotic shock supernatant was supplemented with Tris-HCl pH 8.0 at 20 °C to 50 mM and NaCl to 200 mM, then loaded onto the 5 ml HiTrap Imac HP affinity chromatography column charged with Ni^2+^ ions, which was pre-equilibrated with a 10 column volume (CV) of the buffer A [50 mM Tris-HCl pH 8.0 at 20 °C, 500 mM NaCl]. The HiTrap Imac HP medium was washed with 10 CV of the buffer A. The protein was eluted with 10 CV of the 0-500 mM imidazole gradient in buffer B [50 mM Tris-HCl pH 8.0 at 20 °C, 500 mM NaCl, 500 mM imidazole]. Fractions containing the protein were pooled and dialysed to the 1 × PBS buffer using the Vivaflow 50 Laboratory Cross Flow Cassette [PES] with the Molecular Weight Cut-off (MWCO) of 10 kDa. The dialysed concatemeric protein was concentrated using Vivaspin® Turbo 15 centrifugal concentrators with the PES membrane MWCO of 10 kDa using centrifuges (4000 × g, ca. 15 min, 4 °C) to obtain ca. 1 mg/ml concentration. For the endotoxin removal from the obtained concatemeric protein samples, 1 ml Affi-Prep Polymyxin Resin was used. The resin was regenerated by washing with 0.1 N NaOH with forced flow using a peristaltic pump, then washing with 15 CV of endotoxin-free sterile water. The concatemeric proteins were loaded onto the medium, pre-equilibrated with 10 CV of endotoxin-free PBS buffer. The protein samples were eluted with 6 CV of PBS buffer. The protein fractions were concentrated by lyophilization, then resuspended in the PBS buffer to the target concentration. The purification stages were monitored by the SDS-PAGE and western blotting analysis.

### Western blotting

Purified proteins were separated by SDS-PAGE and electroblotted onto a PVDF membrane [65]. The membrane was probed with murine monoclonal anti-polyHistidine antibodies, conjugated with horseradish peroxidase. A specific protein was visualized by adding a solution of peroxidase substrate as detailed previously [58].

### MCC and CBD fusion proteins interactions

The MCC (100 mg suspended in 100 µl PBS) was spun down and resuspended in 1 × PBS and washed 3 times by sequential centrifugations (10,000 × g/10 min). The fusion proteins purified to homogeneity were suspended in 1 × PBS solution. These two substrates were mixed at a 3 : 1 volume ratio (CBD fusion protein : MCC) and incubated at 4 °C for 18 h. The MCC was then centrifuged and the supernatant collected. The MCC pellet was rinsed with 30 volumes of 1 × PBS solution. Supernatants and pellets were analysed by SDS-PAGE electrophoretic separation. The pellets of MCC complexed with the proteins in the 1 × PBS were observed by TEM microscopy (Tecnai G2 T12 Spirit Bio TWIN firmy FEI Company). Samples were loaded onto a 300 mesh copper grid (Sigma), covered with 2% collodion (Sigma), sprayed with carbon and stained with 2% uranyl acetate (BDH Chemicals), then visualised with a Tecnai G2 Spirit BioTWIN TEM set at 120 kV. Pictures were captured with a Veleta CCD camera.

### Cells isolation and characterization

Human subcutaneous adipose tissue was obtained from a patient undergoing routine surgery at the Department of Oncologic Surgery at the Medical University of Gdansk, Poland. The procedure was approved by the Independent Bioethics Commission for Research of the Medical University of Gdansk (approval number NKBBN/672/2019). The cells were isolated with the procedure described before utilizing collagenase type I [66]. At the 2nd passage cell ‘stemness’ was confirmed by differentiation into adipocytes, osteocytes and chondrocytes [66], and a flow cytometric analysis of characteristic markers: CD73, CD90, and CD105, CD14, CD34, CD45, CD19, and HLA-DR was performed. Data were acquired using the CytoFLEX S Flow Cytometer and analysed by analysis software.

Human Peripheral Blood Mononuclear Cells (PBMCs) were isolated from 10 ml of human EDTA-anticoagulated whole blood which was separated into blood plasma, the buffy coat layer (PBMCs), and red blood cells (RBCs) by density-gradient centrifugation.

### Cell culture

The 46BR.1 N cells (ECCAC) are a skin fibroblasts cell line derived from a patient with hypergammaglobulinemia. The immortalized cell line was obtained by transformation with the pSV3neo plasmid expressing the SV40 antigen [67]. Cells were routinely cultured in Primaria flasks in a DMEM HG (Dulbecco’s Modified Eagle Medium High Glucose - with 4500 mg/l of glucose) medium, supplemented with 10% Fetal Bovine Serum and 100 U/ml penicillin and 100 µg/ml streptomycin in an incubator (37 °C, 5% CO_2_). The culture medium was changed every 2–3 days. The cells were routinely passaged twice a week.

Human adipose derived stem cells (ASCs) were cultured in DMEM LG (Dulbecco’s Modified Eagle Medium Low Glucose) - with 1000 mg/l of glucose medium supplemented with 10% FBS and 100 U/ml penicillin and 100 µg/ml streptomycin. The cells were passaged once a week. Human PBMCs were cultured in an RPMI medium supplemented with 10% FBS and antibiotics.

### Cell proliferation assay (XTT) and cell cytotoxicity assay (LDH)

The XTT (Tetrazolium salt) cell proliferation assay was done using Cell Proliferation Kit II. 46BR.1 N fibroblasts, and ASCs were seeded at a density of 5000 cells per well into 96-well plates in an appropriate medium supplemented with 10% of FBS and 50 U/ml penicillin and 50 µg/ml streptomycin. After 24 h, the media were exchanged for a serum-free DMEM with 50 U/ml penicillin and 50 µg/ml streptomycin and stimulated with an appropriate concentration of MCC. The cells were incubated with MCC for 24 h. After the incubation the plate was centrifuged (1400 rpm, 5 min) and culture supernatant was collected for LDH (Lactate dehydrogenase) analysis. Thereafter, XTT reagent was added and the plates were incubated at 37 °C for 4 h in the presence of 5% CO_2_. The plates were then read using a standard plate reader at OD 490 nm. Cell proliferation was normalized with respect to the non-treated control (100%). LDH cytotoxicity analysis was performed with the Cytotoxicity Detection Kit (Merck) according to the manufacturers’ instructions. Triton X-100 detergent (1%) was used as a positive control for the maximum LDH release (maximum cytotoxicity), while the non - treated cells constituted the negative control (0%).

### Flow cytometric analysis of cell viability

Human ASCs were seeded into 6-well plates (100,000 cells per well) in a DMEM LG medium supplemented with 10% of FBS and 50 U/ml penicillin and 50 µg/ml streptomycin. The next day the medium was changed to a serum-free one and the cells were stimulated with 50, 100, and 200 µg/ml of MCC. Human PBMCs were seeded into 24-well plates (1 million cells per well) in a RPMI medium supplemented with 10% FBS and after 24 h resting, stimulated with 50, 100, and 200 µg/ml of MCC for 24 h.

The effect of the MCC on PBMCs and ASCs viability was assessed using flow cytometry analysis. Cells (ASCs at 2nd passage) were stained by Calcein AM as a measure of enzymatic activity in the live cells, and DAPI (Beckman Coulter, B30437) as a measure of the dead cells with disturbed membrane integrity. Cells were processed with the CytoFLEX S Flow Cytometer. Stained samples (10,000 events) were analysed and compared to the corresponding unstimulated samples.

## Results

### Cellulose hydrolysis

**Fig. 2:**
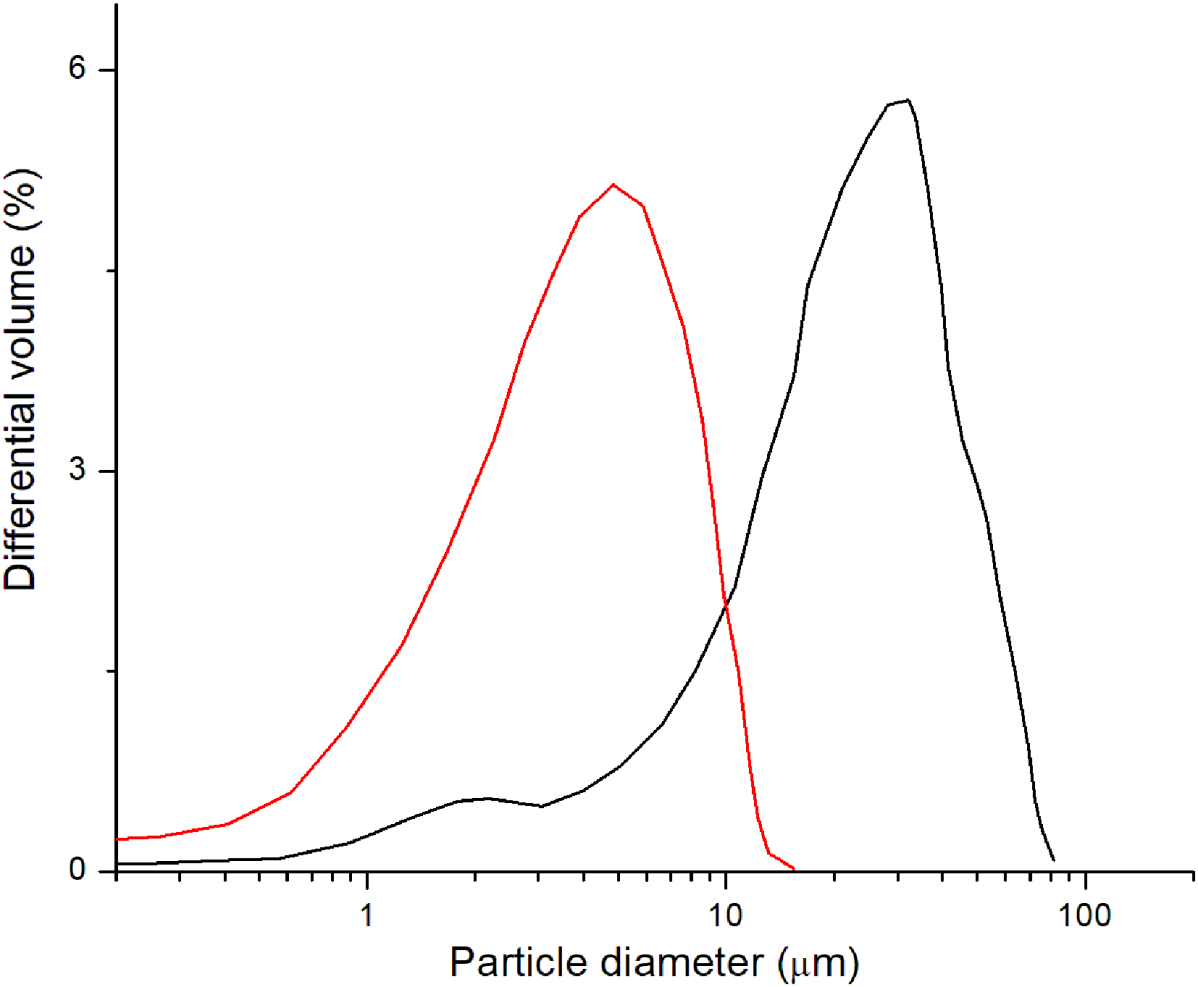
Particle size distribution of microcrystalline cellulose (MCC). Black curve, MCC before hydrolysis by *Aspergillus sp.* cellulase and red curve, MCC after hydrolysis

For starting material, a SigmaCell type 20 MCC was used in all hydrolysis experiments. Suspension of the MCC in a 0.1 M PBS buffer (pH 6.5) forms a thick slurry solution with a strong tendency for sedimentation. The average particle size of the starting MCC material, measured with DLS, was around 22 μm (Fig. 2 - black curve). Hydrolysis of the MCC by *Aspergillus sp.* cellulase is a slow reaction. The reaction was carried out under optimal conditions (PBS buffer, pH 6.5, temp. 30 °C) for *Aspergillus* cellulases described previously [68]. Obtaining particles with diameters of about 3 μm (6-fold reduction in the diameter of the cellulose particle) required running the reaction under optimal conditions for 21 days. One of the main reasons for this is the insolubility of cellulose under reaction conditions, leading to a biphasic reaction. Enzymatic hydrolysis of the MCC by *Aspergillus sp.* cellulase caused an increase in the solution viscosity and a strong decrease in the diameter of the particles to around 3 μm, with a fraction of the particles reaching the nanoparticles size category (Fig. 2 - red curve). The final solution has no tendency for sedimentation.

### DNA FACE™ amplification-expression technology system using pET28a(+) vector-based new variants

In order to facilitate the production of various types of polyepitope proteins, based on the previous general design plan [1, 57–62] we have created new amplification-expression vectors allowing for fusions of potentially therapeutic proteins with the gene encoding the CBD domain. Two novel amplification-expression vectors were designed and constructed, targeting two different locations within expressing cells: the cytoplasm and the periplasmic space. For the latter the MalE leader-coding sequence was incorporated in an arrangement promoting in-frame N-terminal fusions. The original pET28a(+) vector contains the IPTG-inducible T7-lac bacteriophage hybrid promoter and the kanamycin resistance gene, which were retained in the modified construct. Specialized DNA cassettes were designed and chemically synthesized to introduce: (*i*) convergent SapI restriction sites, forming the amplification module; (*ii*) the MalE leader-coding sequence – only in the secretion cassette version; (*iii*) the gene encoding the CBD domain, (*iv)* enterokinase recognition sequence to allow removal of the vector-derived CBD domain from a therapeutic protein with a His6-tag at its C-terminus. The cassettes were cloned into the NcoI and XhoI sites of pET28a(+). The internal SapI restriction site located in the vectors’ backbone was eliminated by site directed mutagenesis prior to cloning. A His6-tag was incorporated for the purpose of using metal affinity chromatography and for expressed protein detection by Western blotting with anti-His6-tag antibodies. The vector pET28a(+) was created with a CBD domain-coding sequence that had been preceded by a MalE leader-coding sequence. Such a secretion leader arrangement enables expressed proteins to be secreted into the periplasmic space. Additionally, the MalE leader in general tends to improve the expression level of recombinant genes, which also applies to concatemeric genes, as we have shown previously [1, 58, 59].

### Designing of a synthetic gene coding for RGD-derived concatemeric protein fusion with the CBD domain, cloning, expression and purification of the protein

**Fig. 3:**
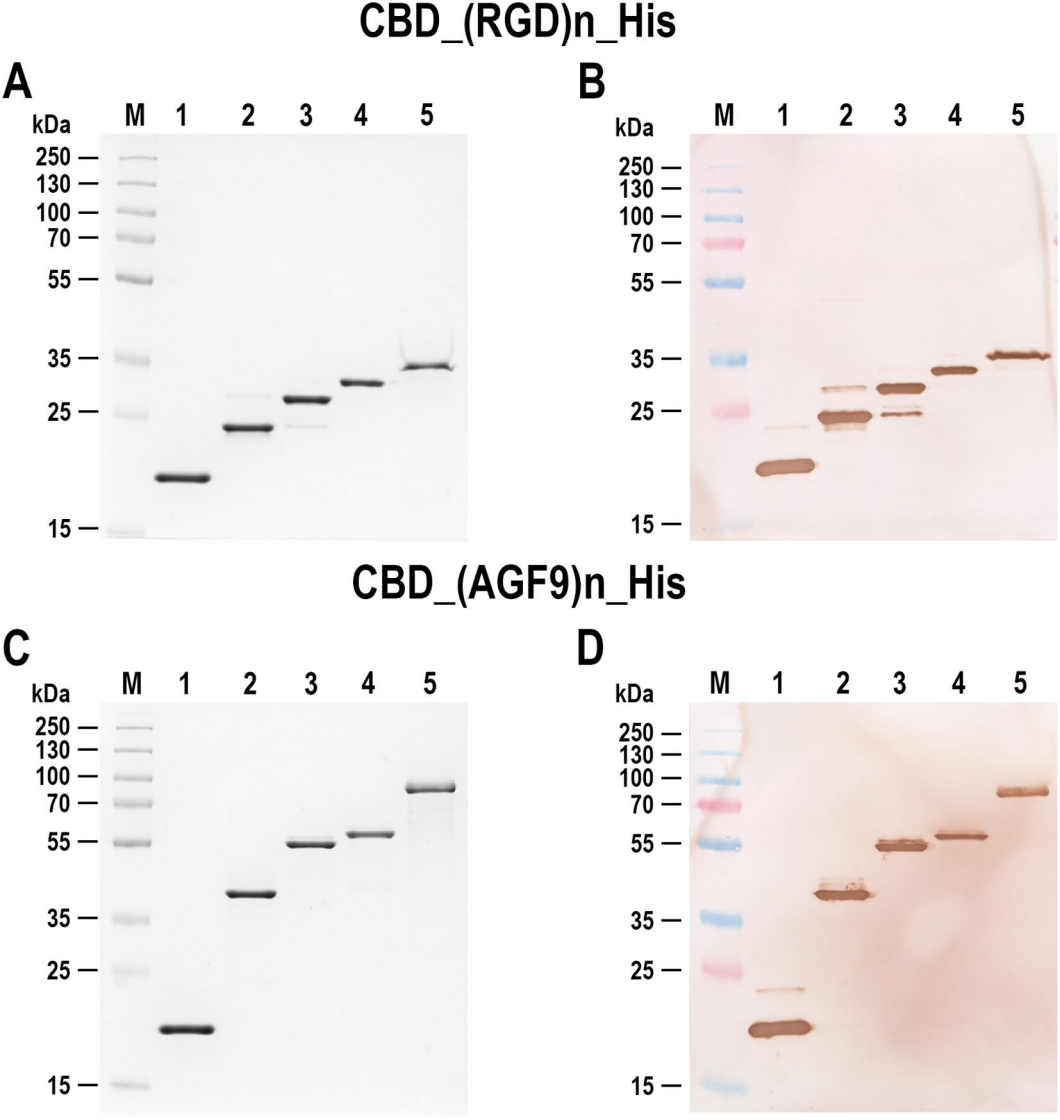
Obtaining the RGD- and AGF-derived concatemeric proteins in fusion with CBD. Panel (**A**) SDS-PAGE analysis of the purified CBD_RGD_X_His protein variants in 12% polyacrylamide gel. M, PageRuler Plus Prestained Protein Ladder; lane 1, CBD_His; lane 2, CBD_RGD_10_His; lane 3, CBD-RGD_20_His; lane 4, CBD-RGD_30_His; lane 5, CBD_RGD_40_His. Panel (**B**) Western blot analysis of proteins described in panel A with the use of anti-polyHistidine antibodies. Panel (**C**) SDS-PAGE analysis of the obtained CBD_AGF_X_His protein variants in 12% polyacrylamide gel. M, PageRuler Plus Prestained Protein Ladder; lane 1, CBD; lane 2, CBD-AGF_10_His; lane 3, CBD-AGF_20_His; lane 4, CBD-AGF_30_His; lane 5, CBD-AGF_40_His. Panel (**D**) Western blot analysis of the proteins described in panel C with the use of anti-polyHistidine antibodies

**Fig. 4:**
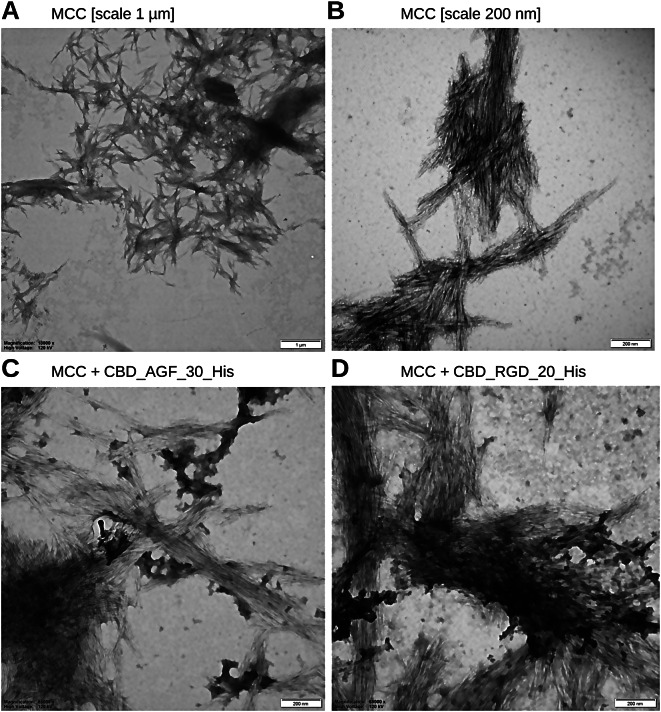
TEM image of the MCC complexes. Panel (**A**) MCC at a magnification 13 000 ×, scale 1 μm. Panel (**B**) MCC at a magnification 68 000 ×, scale 200 nm. Panel (**C**) MCC and CBD_AGF_30_His protein incubated 18 h at 4 °C, then washed 3 times with 1 × PBS and observed with TEM at 68 000 × magnification. Panel (**D**) MCC and CBD_RGD_20_His protein incubated 18 h at 4 °C, then washed 3 times with 1 × PBS and observed with TEM at 68 000 × magnification. Samples loaded onto a 300 mesh copper grid (Sigma), covered with 2% collodion, sprayed with carbon and stained with 2% uranyl acetate and visualised with a Tecnai G2 Spirit BioTWIN TEM set at 120 kV. Pictures were captured with a Veleta CCD camera

A bioactive 3-aa peptide RGD has been selected as a target for concatemeric protein construction [64, 69–72] and it serves as a recognition motif, present in multiple ligands that have substantial potential in tissue regeneration, tumour targeting-based therapy, biological adhesion and as directing theranostic agents [64]. The obtained fusion proteins combined with fragmented MCC particles or nanocellulose particles serve the development of a new strategy of therapeutic bioMCC particles and bionanoparticles as new specialized drugs. The concatemeric DNA cassettes encoding 10, 20, 30, and 40 repeats of the RGD motif, which were previously constructed in plasmids pET28AMP_PhoA_RGD_X [1, 58] were excised by SapI cleavage and cloned into SapI cleaved pET28_delSapI_CBD_His and pET28_delSapI_MalE_CBD_His amplification-expression vectors, forming fusions of concatemeric proteins with a CBD domain. The resultant recombinant *E. coli* BL21(DE3) ClearColi clones were analysed using the colony PCR, SapI restriction mapping and DNA sequencing. The presence of concatemeric genes, containing 10, 20, 30, and 40 repeats of the RGD fusions with the CBD domain were confirmed. For gene expression experiments, bacterial clones producing concatemeric proteins with 10, 20, 30, 40 repeats of the RGD fusion with the CBD domain were selected from pET28_delSapI_MalE_CBD_X_His expression constructs. The resulting concatemeric RGD motif-CBD fusion proteins were purified to homogeneity (Fig. 3A and B) and their interaction with MCC was confirmed (Fig. 4C and D). Constructs containing multiple repeats of the AGF motif peptide were obtained using the same strategy as for the RGD peptide (Fig. 3C and D).

### Biological effects of MCC on human cells

The biological safety of MCC was analysed on human cells with the use of the XTT proliferation test, LDH cytotoxicity assay and viability analysis with flow cytometry.

The ‘stemness’ phenotype of human ASCs was confirmed before MCC experiments (Additional file 1). Analysis of adipose-derived stem cells (ASCs) was performed according to the International Society for Cellular Therapy (ISCT) guidelines. The analysed cells were plastic-adherent, showed an ability for multi-lineage differentiation (osteogenic, adipogenic and chondrogenic), and showed key surface markers during a flow cytometry analysis. Isolated ASCs demonstrated the immunophenotypic characteristics of MSCs, with an expression of surface markers CD73, CD90, and CD105 (≥ 90%), and a lack of expression of the hematopoietic markers CD14, CD34, CD45, CD19, and HLA-DR (≤ 2%).

**Fig. 5:**
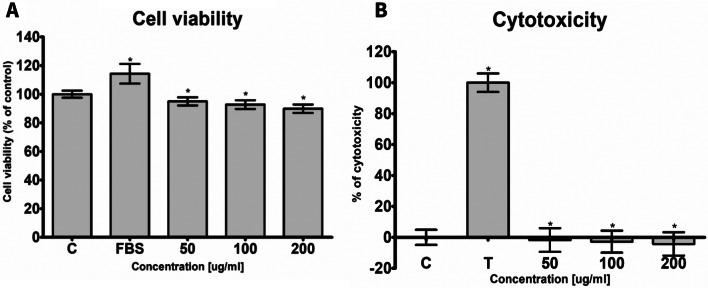
The effect of MCC on the proliferation and MCC cytotoxicity on fibroblast 46BR.1 N. Panel (**A**) The effect of MCC on the proliferation of 46BR.1 N fibroblasts. Panel (**B**) MCC cytotoxicity on the human fibroblast 46BR.1 N cell line. The results are presented as mean ± SEM and come from 4 independent experiments performed in quadruple. An asterisk (*) shows the statis-tically significant differences compared to the non-treated control, Mann Whithey U-test, *n* = 16; C, control, cells cultured in medium without serum; FBS, positive control in XTT assay, cells cultured in a medium containing 10% FBS; T, positive control in the LDH test, cells treated with 1% triton X

**Fig. 6:**
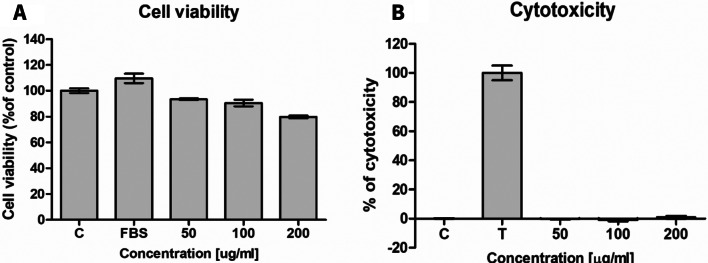
The effect of MCC on the proliferation and MCC cytotoxicity on human adipose-derived stem cells. Panel (**A**) The effect of MCC on the proliferation of human adipose-derived stem cells. Panel (**B**) MCC cytotoxicity on human adipose-derived stem cells (*n* = 4 technical replications). C, control, cells cultured in medium without serum; FBS, positive control in XTT assay, cells cultured in a medium containing 10% FBS; T, positive control in the LDH test, with cells treated with 1% triton X

In the LDH assay we did not observe cytotoxicity of MCC towards human 46BR.1 N fibroblasts and ASCs (Figs. 5B and 6B). The XTT analysis revealed a slight (5–10%) inhibition of the proliferation of 46BR.1 N cells after stimulation with all the tested concentrations of MCC (Fig. 5A). Similar results were obtained for ASCs stimulated with 50 and 100 µg/ml of MCC. The strongest inhibition (up to 20% compared to the control) was observed for ASCs stimulated with MCC in the concentration of 200 µg/ml (Fig. 6A). However, we did not observe significant changes in cell morphology after the stimulation with MCC (Additional file 2). Flow cytometric analysis of the cell viability showed no harmful influence of MCCs on PBMC and ASCs (Additional files 3 and 4). Only for the ASCs’ number of viable cells (calcein+) was there a slight decrease for the highest MCC concentration (99.7% for 50 µg/ml MCC; 99.6% for 100 µg/ml, and 94.9% for 200 µg/ml).

### Stability of the CBD_AGF_30_His and CBD_RGD_20_His proteins in the PBS buffer and human serum

**Fig. 7:**
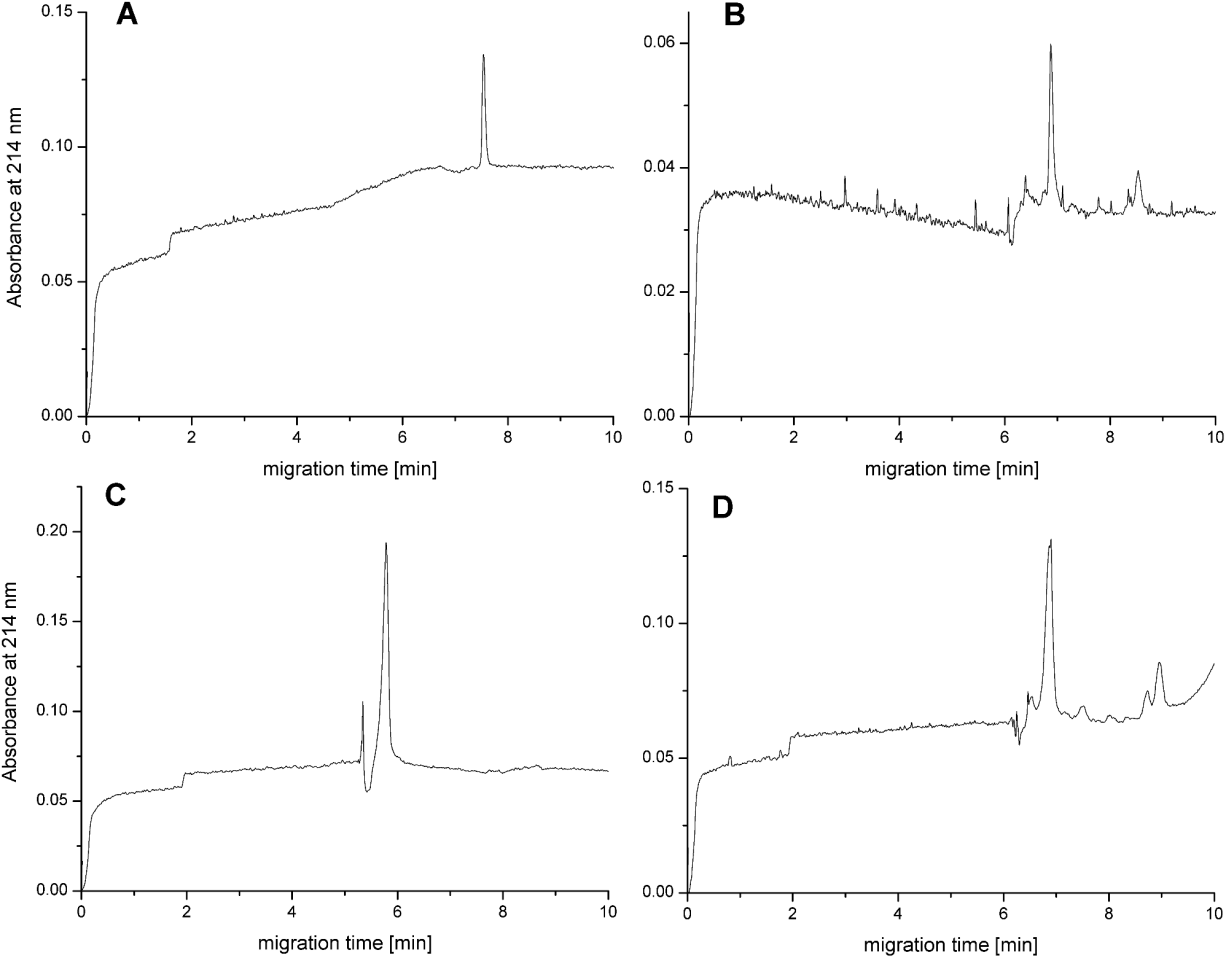
The CE electropherograms of CBD_AGF_30_His and CBD_RGD_20_His stability in PBS and serum. Panel (**A**) Representative CE electropherograms of CBD_AGF_30_His proteins stability in 0.1 × PBS pH 7.2. Panel (**B**) Representative CE electropherograms of CBD_AGF_30_His proteins stability in serum. Panel (**C**) Representative CE electropherograms of CBD_RGD_20_His proteins stability in 0.1 × PBS pH 7.2. Panel (**D**) Representative CE electropherograms of CBD_RGD_20_His proteins stability in serum

The CE analysis of the CBD_AGF_30_His and CBD_RGD_20_His proteins showed that they were highly homogeneous and migrated as single peak in the capillary under the analysis conditions used (Fig. 7A,C).

**Fig. 8:**
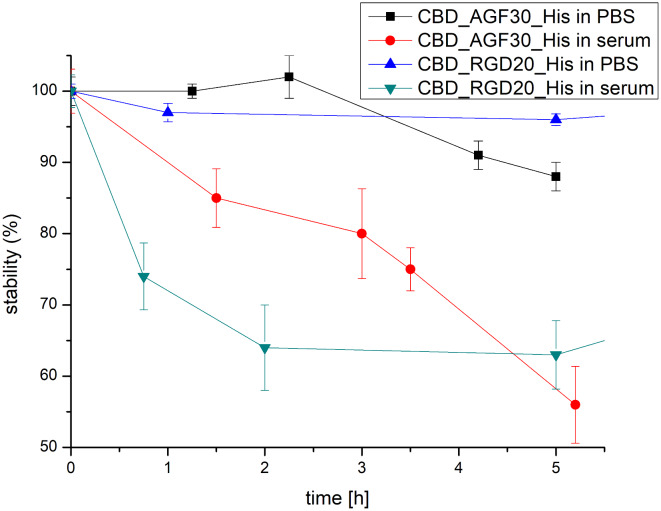
CBD_AGF_30_His and CBD_RGD_20_His proteins stability derived from the CE analysis data

Only in the case of CBD_RGD_20_His protein was an additional peak of much lower intensity characterised by a slightly shorter migration time in relation to the main peak observed (Fig. 7C). At 37 °C, both proteins were stable for at least several hours (Fig. 8).

A slight decrease in peak intensity (in the order of 10–15%) was only observed for the CBD_AGF_30_His protein after 4–5 h of incubation. This demonstrates the stability of both proteins in pH-neutral aqueous solutions at elevated temperature. Following, CE analysis of CBD_AGF_30_His and CBD_RGD_20_His proteins stability in human serum was performed. A human serum was chosen as a model physiological fluid useful for characterising protein stability under physiological-like conditions. Preliminary CE analysis of a human plasma sample showed that albumin, which is the main plasma protein fraction, was not visible under the separation conditions used, and that the signals from the other plasma components were of low intensity and did not significantly interfere with those from the proteins under study (data not shown). The peak of the latter was dominant in the mixture of plasma with the test proteins. Typical electropherograms of the analysis of the mixture of test proteins in human plasma are shown in Fig. 7B, D. In the case of the CBD_AGF_30_His protein, only a slight shortening of the migration time of the proximal peak was observed, compared to the signal obtained in the PBS buffer (Fig. 7A, B). In contrast, for the CBD_RGD_20_His protein, a clear increase in the migration time of its peak was observed after mixing the sample with human plasma (Fig. 7B, D). The small additional peaks seen in these electropherograms, both with longer and shorter migration times in relation to the signals of the proteins studied, originate both from the (mainly protein) components of the plasma and the degradation products of the proteins studied, which were not further investigated.

The CE analyses showed that both proteins undergo progressive time-dependent degradation in human plasma (Fig. 8). This process was observed and characterised by a decrease in the intensity of the peak corresponding to the protein under study. In the case of the CBD_RGD_20_His protein, the dynamics of this process were much greater than for the CBD_AGF_30_His protein. After 1 h, about 75% of the initial amount of CBD_RGD_20_His was observed, while for the CBD_AGF_30_His protein this value was over 90%. The degradation profile obtained with the CE method shows that the degradation of the CBD_RGD_20_His protein was much faster than that of the CBD_AGF_30_His protein. Only after about 5 h did the content of the unhydrolyzed proteins approach each other and reach a level of about 55–60% of their initial amount. This demonstrates the moderate stability of the studied proteins in plasma and the much faster rate of hydrolysis of the CBD_RGD_20_His protein compared to CBD_AGF_30_His within the first few hours of contact with plasma proteins.

## Discussion

In this article, we present: (i) a new method for the conversion of available MCC preparations to forms of severalfold smaller size by a series of enzymatic reactions in specific sequential order and (ii) a novel type of hybrid MCC-protein particles, approaching the nano size range, obtained by affinity complexing via CBD. The enzymatic hydrolysis of MCC by *Aspergillus sp.* cellulase proved to be an effective process leading to a significant reduction of its particle size. As a result of hydrolysis, fragmented MCC was obtained, the particle diameter of which was more than six times and the volume about 230 times smaller than the starting MCC material. The obtained partially hydrolysed MCC were highly homogeneous as shown by the particle size distribution analysis. Here we focused on the properties of MCC as a carrier of therapeutic proteins. CBD proteins are sequences that have the ability of specific binding to cellulose. This property allows them to interact with nanocellulose, which has applications in many fields, including biotechnology, medicine, and the food industry. The binding of CBD to nanocellulose can significantly increase the affinity and performance of nanocelluloses as a carrier, biomaterial, or platform for various applications. In the context of nanocellulose functionalization, CBD proteins are promising tools that open a wide range of applications. Research confirming these applications provides a solid basis for using the nanocellulose and CBD protein combinations to improve their functionality and use in various industrial and scientific fields. For this purpose, we obtained recombinant proteins fused with a CBD tag that interacts specifically with MCC. The proteins selected for fusions with CBD were highly reengineered, or ‘artificial’ (= not existing in Nature), which carried condensed functionalisation in the form of concatemers. These were composed of repetitive, bioactive epitopes, oriented in organised head-to-tail fashion, expressed from continuous ORFs, constructed using DNA FACE™ technology [1, 57–62]. For the purpose of this work, new amplification-expression vectors were constructed, following the general design of DNA FACE™ technology, which were dedicated to concatemeric ORFs fusions with CBD and possessing the following features: (i) an IPTG-inducible promoter for cytoplasmic expression at temperatures from 28 to 37 °C, which can help maintain the polyepitope solubility of proteins, as well as improve the transcription of GC-rich genes, characterised by a high level of gene expression; (ii) the lac repressor gene is on the same vector backbone; (iii) IPTG inducible T7-lac promoter; (iv) the CBD cassette allowing for the fusion with concatemeric protein; (v) removable CBD domain by an enterokinase or TEV protease recognition site and (vi) IPTG-induced expression of the T7-lac promoter in combination with MalE-directed secretion of fusion concatemeric proteins into the periplasm. This strategy will be also useful with ‘toxic’ concatemeric proteins, proteins that require disulfide bond formation. For the construction of CBD fusion proteins, we focused on the concatemeric proteins RGD and AGF, because, in our previous studies, these proteins showed a lack of immunogenicity, as well as low allergenic potential and low cytotoxicity while having pro-proliferative and pro-migratory effects. They are considered as potential wound healing stimulants [1]. In this work, the CBD domain with the His6-tag and 8 fusion proteins were obtained: (i) with an RGD peptide - CBD_RGD_10/20/30/40_His and (ii) with the AGF9 peptide - CBD_AGF_10/20/25/40_His. All the proteins were obtained by inducing the expression of fusion genes directing them into the periplasmic space. High yields of proteins were obtained and their identity was confirmed by the Western blot with anti-His antibodies. Out of those for MCC interaction studies, CBD_RGD_20_His and CBD_AGF_30_His were selected, due to their highest yields and purity. The results show a strong interaction of the proteins with MCC. As confirmed by SDS-PAGE and TEM analysis, washing of MCC with immobilised proteins does not cause their release from complexes with MCC. In addition, a study of the interaction of biomolecules on the Monolith X Nanotemper machine indicated a strong interaction at the nanomolar level. Incubation and washing was carried out in a 1 × PBS solution as it closely resembles the physiological environment. However, we encountered problems with salt precipitation in these formulations that interfered with the TEM analysis; nevertheless the results allowed for confirmation of the complexes formation. The analysis could not be performed in water alone, as the interaction between MCC and fusion proteins requires a buffered solution with a certain level of salts present to maintain the native conformation and solubility of the CBD. Thus the obtained MCC particles showed a stable affinity for CBD and its fusions with proteins, which allows their potential use as a carrier of bioactive proteins for a variety of applications, exemplified by: medically as novel drugs or wound healing preparations, industrial biocatalysts, adsorption materials, environmental detoxification, among others. Furthermore, the use of specialised ‘artificial’ concatemeric proteins immobilised on microparticle and nanoparticle carriers may have an increased activity or adapt to a specialised application. Such a strategy may enhance the potential therapeutic or biotechnological effect of MCC-complexed proteins. However, it is important to analyse the biological safety of MCC and nanocellulose carriers to exclude any harmful effects (e.g. toxicity) to human cells. The biological safety of MCC was assessed on three types of cells: a transformed line of human fibroblasts, human adipose-derived stem cells (ASCs) and human lymphocytes. It was proved that MCC is safe for human cells and causes only very low cytotoxicity assessed by using standard toxicity and proliferation tests based on colorimetric methods. Additionally, the high viability of the primary cell lines stimulated by MCC using more advanced flow cytometry methods (DAPI and Calcein staining techniques) was confirmed. Observed slight inhibition of proliferation may be related to the physical effect of MCC on cells in cell culture conditions, as MCC is not a water-soluble material. We decided to use stem cells obtained from fat tissue due to their tremendous therapeutic potential. They have regenerative and immunoregulatory properties. ASCs are the subject of intensive research, both in basic science and clinical trials. MCC, which can be used as a scaffold and drug carrier, is a promising tool for tissue engineering and regenerative medicine. Therefore safety research presented in this study is an important contribution to confirm practical applications of the developed novel biomaterials. During this study, no biological effects of MCC-complexed therapeutic proteins were shown, as these are outside the scope of this work and will be further evaluated in a separate study.

Nanocellulose carriers show many advantages for concatemeric therapeutic proteins, as confirmed by scientific studies. Here are some advantages of these carriers in the context of concatemeric therapeutic proteins: (i) protein stability and protection: nanocellulose can provide stability and protection for concatemeric therapeutic proteins, preserving them from enzymatic degradation or other factors that can negatively affect their therapeutic activity [33]; (ii) potential for drug delivery: nanocellulose can be used as a carrier to deliver concatemeric therapeutic proteins to specific sites in the body, which can enhance their bioavailability and therapeutic efficacy [25]; (iii) biocompatibility: cellulose nanocarriers are often considered biocompatible materials, meaning that they can limit potential immune reactions or toxic side effects during concatemeric protein therapy [32]; (iv) regulated drug release: nanocellulose carriers can be tailored for the controlled release of concatemeric therapeutic proteins, allowing the drug concentration to remain stable in the body for longer periods of time, which is beneficial for long-term therapies [25, 28]. The use of nanocellulose carriers for concatemeric therapeutic proteins is a promising area of research due to their stabilizing properties, drug delivery capability, and biocompatibility. Research confirming these advantages provides a sound basis for the therapies using concatemeric proteins in combination with nanocellulose carriers.

## Conclusions

1. Two novel amplification-expression DNA FACE™ technology vectors were constructed for fusions of CBD with concatemeric proteins directing expression to the cytoplasm or periplasm.
2. Micrometre-diameter MCC particles were efficiently generated by a novel approach to enzymatic hydrolysis using thermostable *Aspergillus sp.* cellulases.
3. Two series of CBD fusions were constructed and the proteins purified: (i) with RGD motif, amplified 10, 20, 30, 40 times and (ii) AGF with RGD motif, amplified 10, 20, 30, 40 times.
4. The method for the conversion of commercial MCC to severalfold smaller particle size MCC and further to nanocellulose was developed.
5. Novel types of hybrid bioMCC were developed, exposing fusion CBD-concatemeric proteins via CBD-MCC affinity interaction.
6. Safety assessment of the constructed MCC particles has shown no negative effects on cultured human cells.

### Electronic supplementary material

Below is the link to the electronic supplementary material.

**Supplementary Material 1:** Supporting informations for publication

**Supplementary Material 2:** Raw images

## Data Availability

All data generated or analysed during this study are included in this published article. The datasets used and/or analysed during the current study are available from the corresponding author on reasonable request. Supplementary Materials: The following supporting information can be downloaded: Figure S1: The confirmation of ASCs immunophenotype. A flow cytometric analysis of the key positive and negative surface markers (according to ISCT guidelines) was conducted; Figure S2: Morphology of fibroblast cell lines. The cells (46BR.1 N) were stimulated by MCC (24 h); Figure S3: Effect of the MCC on PBMC viability. This was checked by DAPI ( DAPI+ dead cells; B-F) and Calcein AM (alive cells; G-I) staining. Figure S4: Influence of the MCC on ASC viability. This was checked by DAPI (DAPI+ dead cells; B-F) and Calcein AM (Calcein+ alive cells; G-I) staining; Figure S5: New plasmid vector maps; Figure S6: New plasmid maps with AGF_poliepitopic and RGD_poliepitopic proteins.

## Declarations

### Patents

A polish patent application has been filed with the title: “A method for the preparation of sub-micrometer particle diameter microcelulose, submicrometer particle diameter microcelulose its use as a biocarrier for fusion proteins, linked to the cellulase binding domain of the microcelulose.” The number [WIPO ST 10/C PL446913] has been assigned.

## Abbreviations

CBD: cellulose binding domain
CE: capillary electrophoresis
DLS: dynamic light scattering
FBS: fetal bovine serum
LDH: lactate dehydrogenase
MCC: microcellulose
ORF: open reading frame
PBMCs: Human Peripheral Blood Mononuclear Cells
TEM: Transmission electron microscopy
