# Supplementary material for: Development of hybrid biomicroparticles: cellulose exposing functionalized fusion proteins
Source: Microb Cell Fact. 2024 Mar 14;23:81. doi: 10.1186/s12934-024-02344-x (PMC10938831; doi:10.1186/s12934-024-02344-x)
Supplement: Supplementary file 1 — Supplementary Material 1: Supporting informations for publication [file 12934_2024_2344_MOESM1_ESM.pdf]

SUPPORTING INFORMATION

Development of hybrid biomicroparticles: cellulose exposing functionalized fusion proteins

Joanna Żebrowska[1,2†], Piotr Mucha[3†], Maciej Prusinowski[1], Daria Krefft[1,2], Agnieszka Żylicz-Stachula[1,2], Milena Deptuła[4], Aneta Skoniecka[4], Agata Tymińska[4], Małgorzata Zawrzykraj[5], Jacek Zieliński[6], Michał Pikuła[4], Piotr M. Skowron[1,2]*

[1] Department of Molecular Biotechnology, Faculty of Chemistry, University of Gdansk, Gdansk, 80-308, Poland

[2] BioVentures Institute Ltd., Poznan, 60-141, Poland

[3] Department of Molecular Biochemistry, Faculty of Chemistry, University of Gdansk, Gdansk, 80-308, Poland Laboratory of Tissue Engineering and Regenerative Medicine, Division of Embryology, Faculty of Medicine, Medical University of Gdansk, Gdansk, 80-211, Poland

[4] Laboratory of Tissue Engineering and Regenerative Medicine, Division of Embryology, Faculty of Medicine, Medical University of Gdansk, Gdansk, 80-211, Poland

[5] Division of Clinical Anatomy, Faculty of Medicine, Medical University of Gdansk, Gdansk, 80-211, Poland

[6] Department of Oncologic Surgery, Faculty of Medicine, Medical University of Gdansk, Gdansk, 80-211, Poland

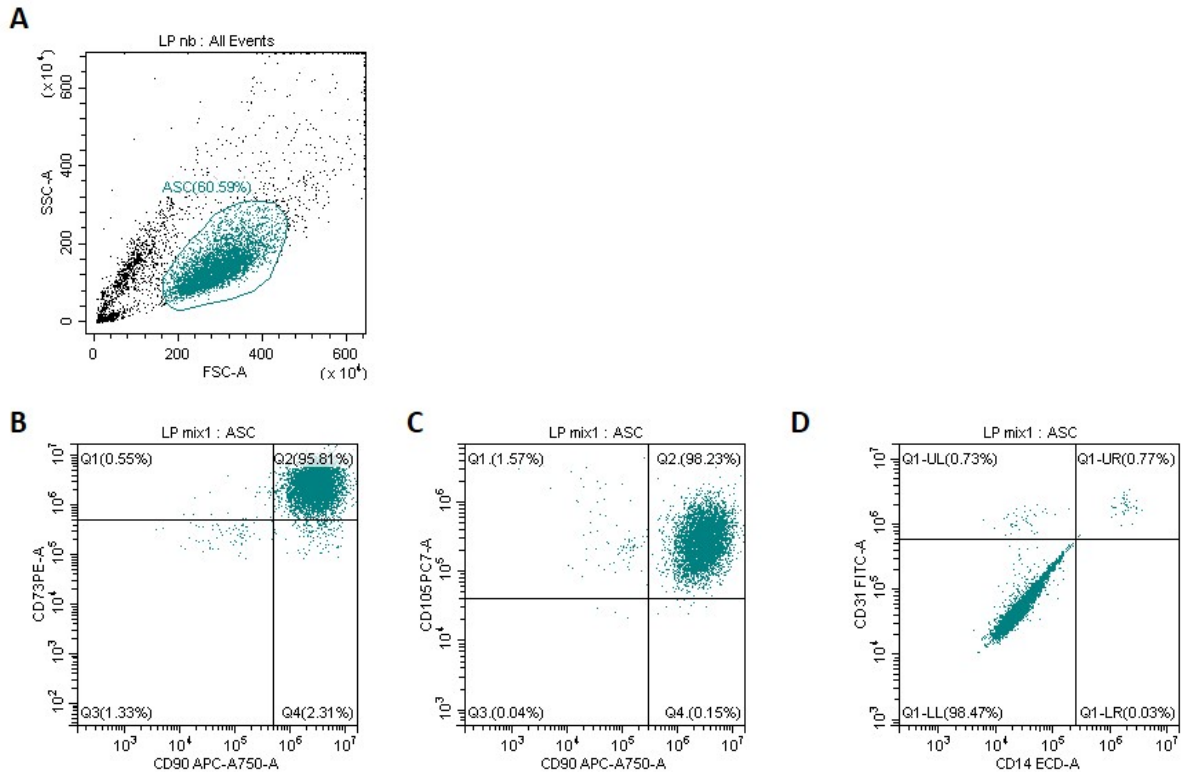

Figure 1S. The confirmation of ASCs immunophenotype. A flow cytometric analysis of the key positive and negative surface markers (according to ISCT guidelines) was conducted. (A), Representative dot plots with gated adipose-derived stem cells (ASC); (B), ASC cells with positive surface markers CD73+, CD90+ (95,81%); (C), and CD105+, CD90+ (98,23%); and (D), negative surface markers CD31-, CD14- (98,47%).

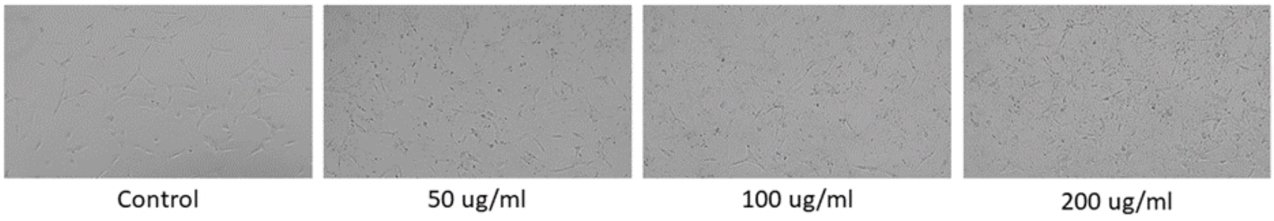

Figure 2S. Morphology of fibroblast cell lines. The cells (46BR.1N) were stimulated by MCC (24 h).

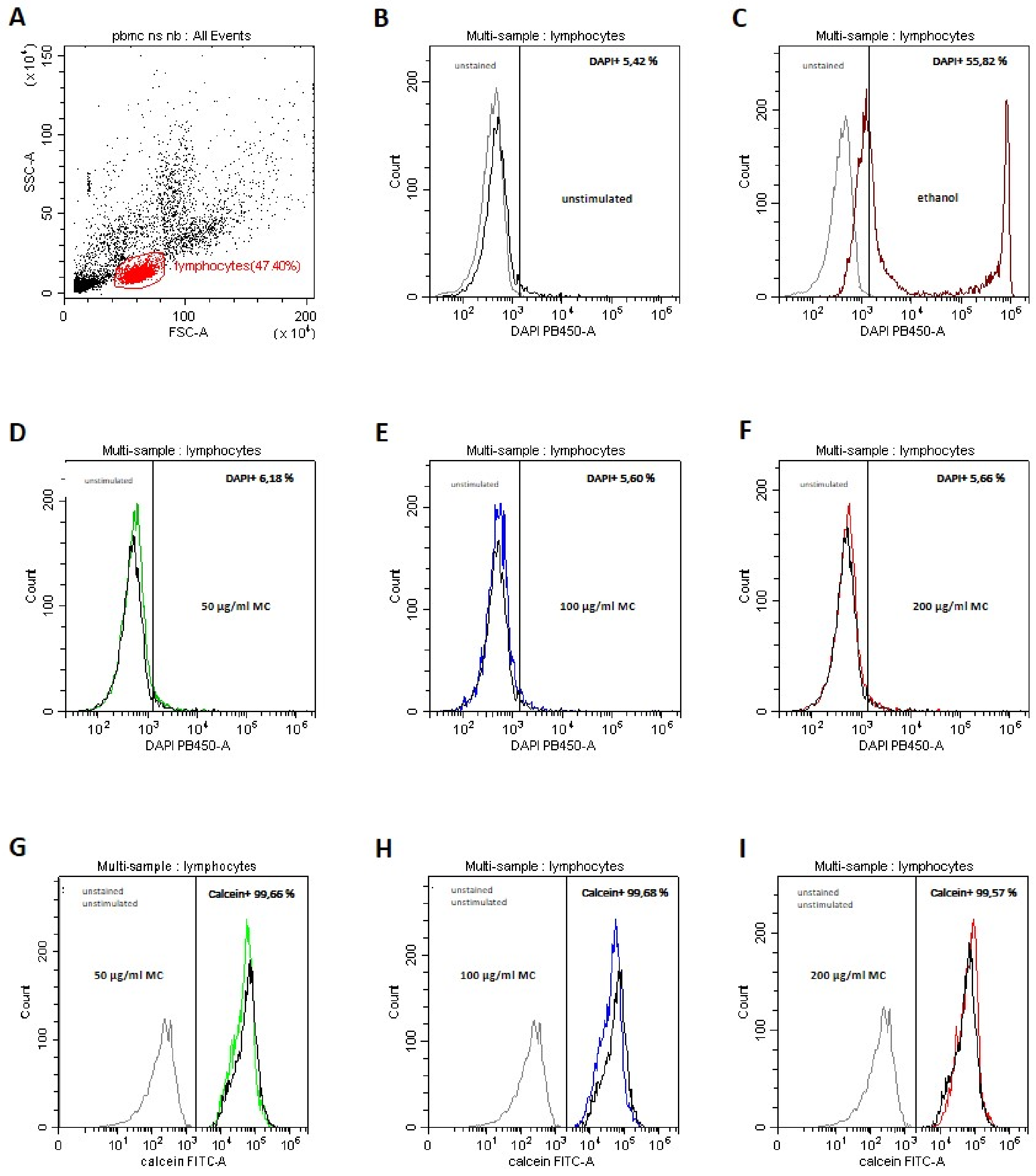

Figure 3S. Effect of the MCC on PBMC viability. This was checked by DAPI (DAPI+ dead cells; B-F) and Calcein AM (alive cells; G-I) staining. Representative dot plot of PBMC with gated lymphocytes (A). Representative overlay histograms analysis of unstained and unstimulated cells (B), and DAPI-stained: ethanol-treated (C) as a positive control with dead cells. On histograms D-F, cells were stimulated by MCC in different concentrations (50 µg/ml – D (green line), 100 µg/ml – E (blue line), 200 µg/ml – F (red line)), and compared to the corresponding unstimulated sample. The histograms G-I show alive cells stained with Calcein AM stimulated by MCC (50 µg/ml – G (green line), 100 µg/ml – H (blue line), 200 µg/ml – I

(red line)), compared with the unstained and unstimulated sample. Obtained values refer to the gated lymphocytes area.

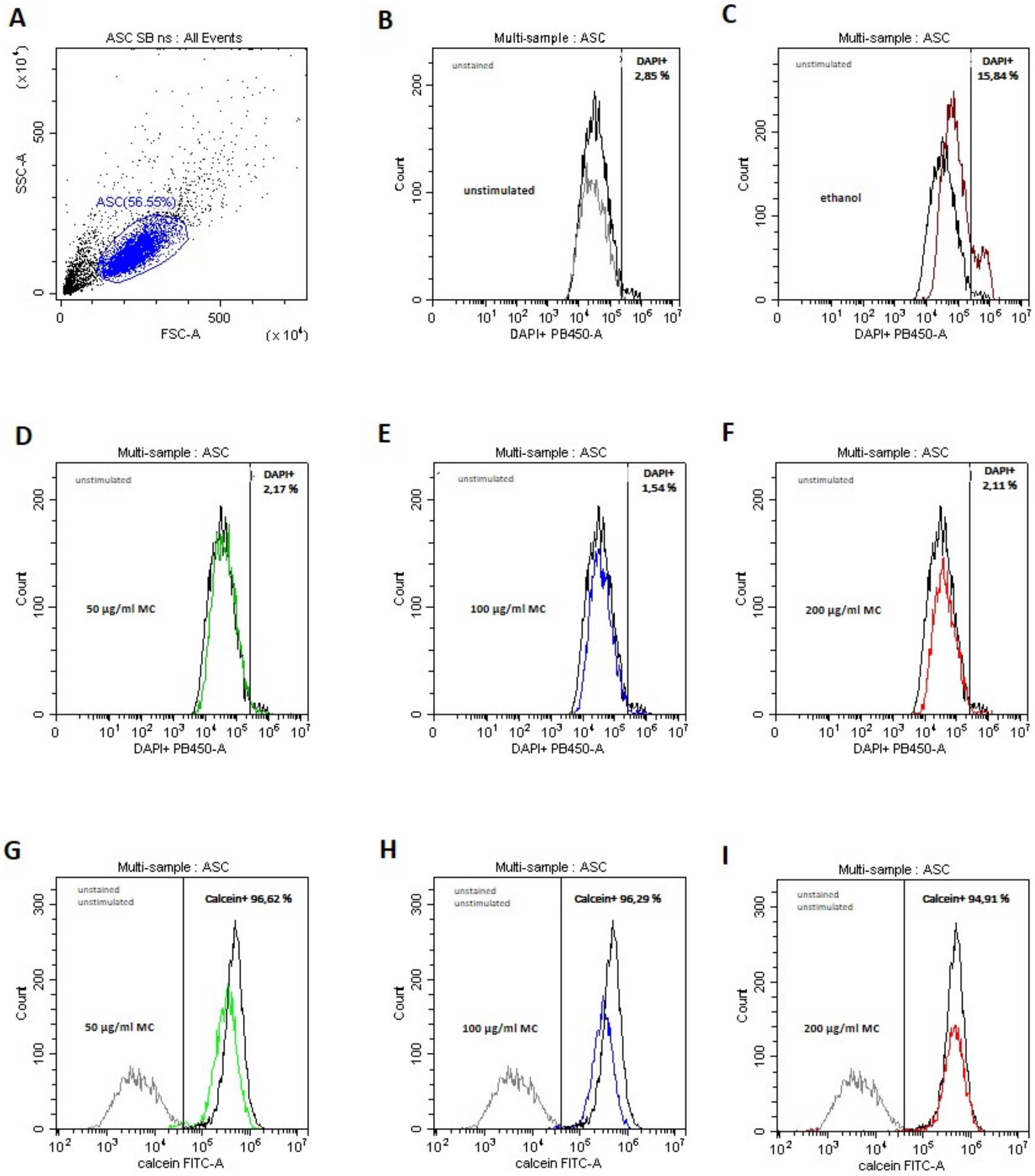

Figure 4S. Influence of the MCC on ASC viability. This was checked by DAPI (DAPI+ dead cells; B-F) and Calcein AM (Calcein+ alive cells; G-I) staining. Representative dot plot of gated ASCs (A). Representative overlay histograms analysis of unstained cells (B), and DAPI-stained: ethanol-treated (C) as a positive control with dead cells. On histograms D-F, cells were

stimulated by MCC in different concentrations (50 µg/ml – D (green line), 100 µg/ml – E (blue line), 200 µg/ml – F (red line)), and compared to the corresponding unstimulated sample. The histograms G-I show alive cells stained with Calcein AM –stimulated by MCC (50 µg/ml – G (green line), 100 µg/ml – H (blue line), 200 µg/ml – I (red line)), compared with the unstained and unstimulated samples. Obtained values refer to the gated ASCs area.

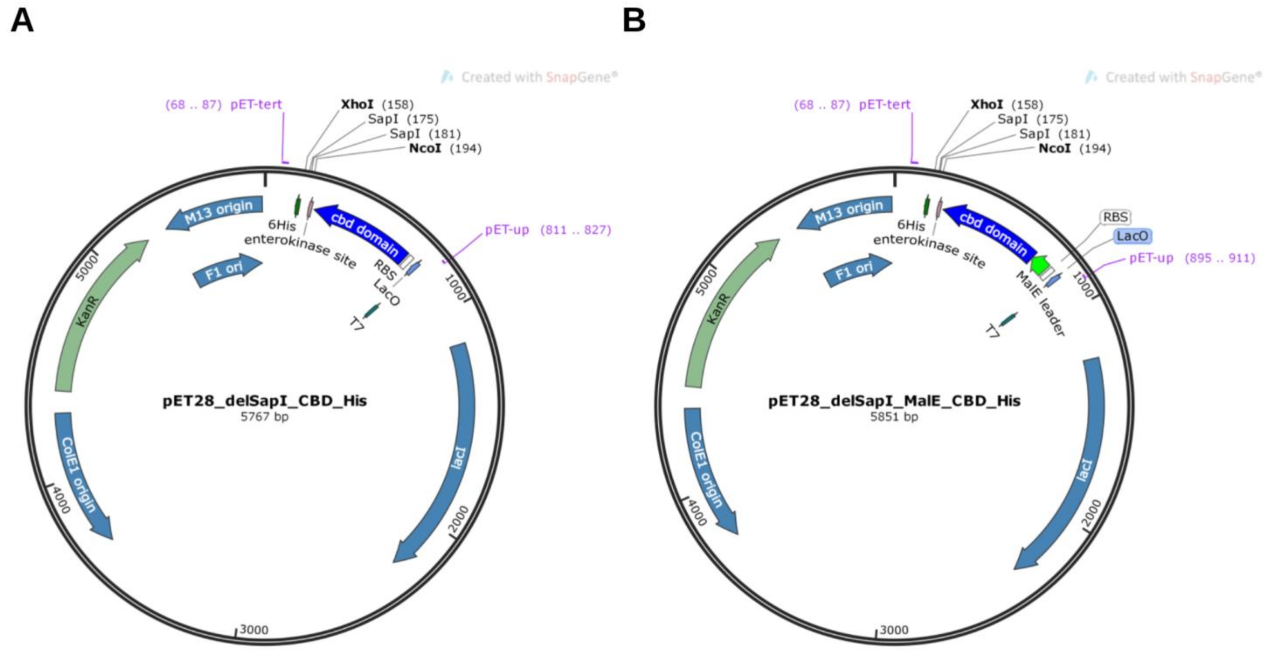

Figure 5S. New plasmid vector maps. Panel A. pET28_delSapI_CBD_His**.** Panel B. pET28_delSapI_MalE_CBD_His.

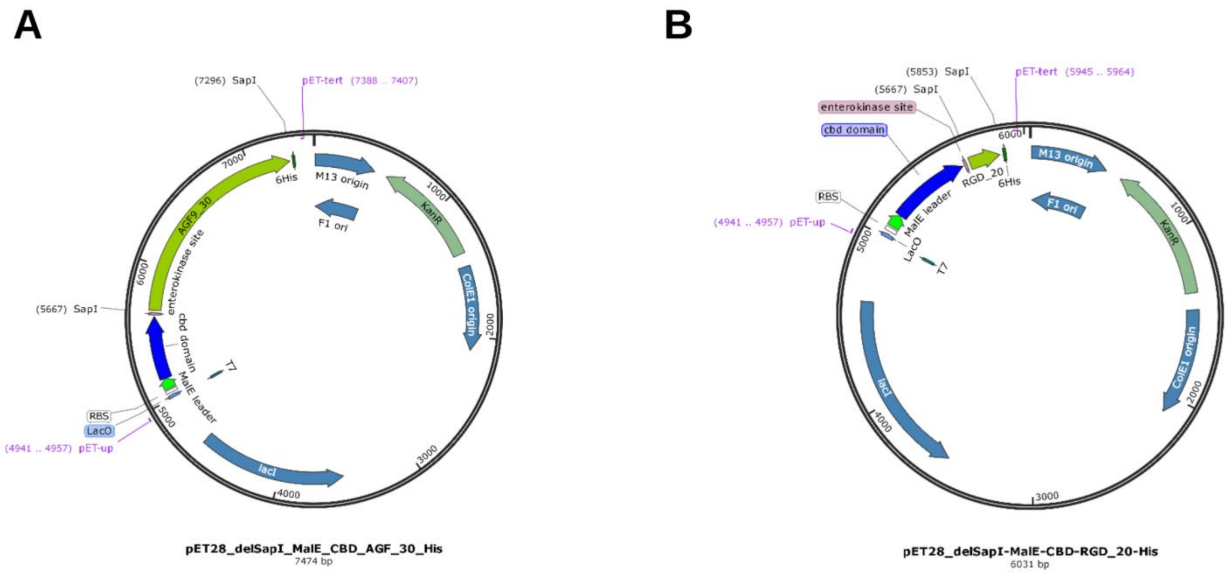

Figure 6S. New plasmid maps with AGF_poliepitopic and RGD_poliepitopic proteins. Panel A. Map of pET28_delSapI_MalE_CBD_AGF_30_His**.** Panel B. Map of pET28_delSapI_MalE_CBD_RGD_20_His.
