# Supplementary material for: Development of hybrid biomicroparticles: cellulose exposing functionalized fusion proteins
Source: Microb Cell Fact. 2024 Mar 14;23:81. doi: 10.1186/s12934-024-02344-x (PMC10938831; doi:10.1186/s12934-024-02344-x)
Supplement: Supplementary file 2 — Supplementary Material 2: Raw images [file 12934_2024_2344_MOESM2_ESM.docx]

**SUPPORTING INFORMATION**

**Development of hybrid biomicroparticles: cellulose exposing functionalized  fusion proteins**

*Joanna Żebrowska^1,2†*^, Piotr Mucha^3†^, Maciej Prusinowski^1^, Daria Krefft^1,2^, Agnieszka Żylicz-Stachula^1,2^, Milena Deptuła^4^, Aneta Skoniecka^4^, Agata Tymińska^4^, Małgorzata Zawrzykraj^5^, Jacek Zieliński^6^, Michał Pikuła^4^, Piotr M. Skowron^1,2^*

^1^ Department of Molecular Biotechnology, Faculty of Chemistry, University of Gdansk, Gdansk, 80-308, Poland

^2^ BioVentures Institute Ltd., Poznan, 60-141, Poland

^3^ Department of Molecular Biochemistry, Faculty of Chemistry, University of Gdansk, Gdansk, 80-308, Poland Laboratory of Tissue Engineering and Regenerative Medicine, Division of Embryology, Faculty of Medicine, Medical University of Gdansk, Gdansk, 80-211, Poland

^4^ Laboratory of Tissue Engineering and Regenerative Medicine, Division of Embryology, Faculty of Medicine, Medical University of Gdansk, Gdansk, 80-211, Poland

^5^ Division of Clinical Anatomy, Faculty of Medicine, Medical University of Gdansk, Gdansk, 80-211, Poland

^6^ Department of Oncologic Surgery, Faculty of Medicine, Medical University of Gdansk, Gdansk, 80-211, Poland


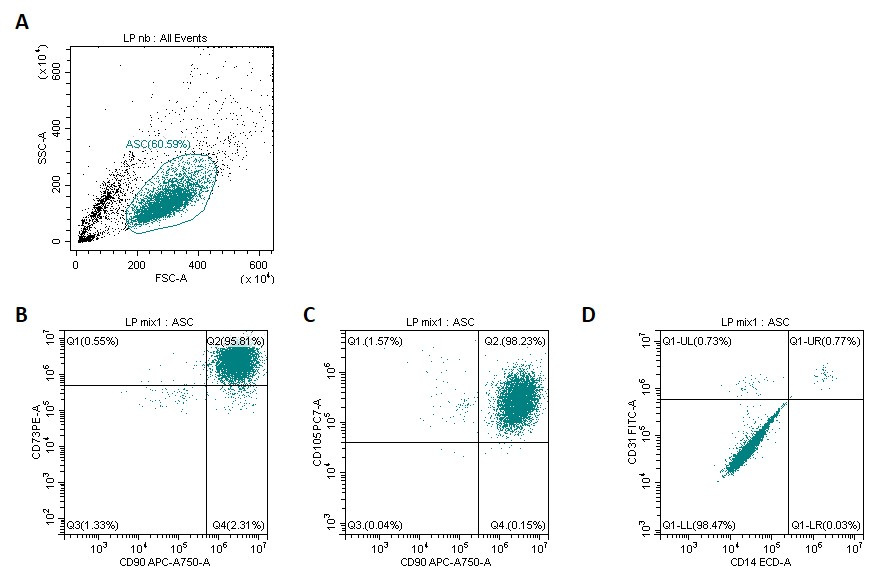


**Figure 1S.** The confirmation of ASCs immunophenotype. A flow cytometric analysis of the key positive and negative surface markers (according to ISCT guidelines) was conducted. (A), Representative dot plots with gated adipose-derived stem cells (ASC); (B), ASC cells with positive surface markers CD73+, CD90+ (95,81%); (C), and CD105+, CD90+ (98,23%); and (D), negative surface markers CD31-, CD14- (98,47%).


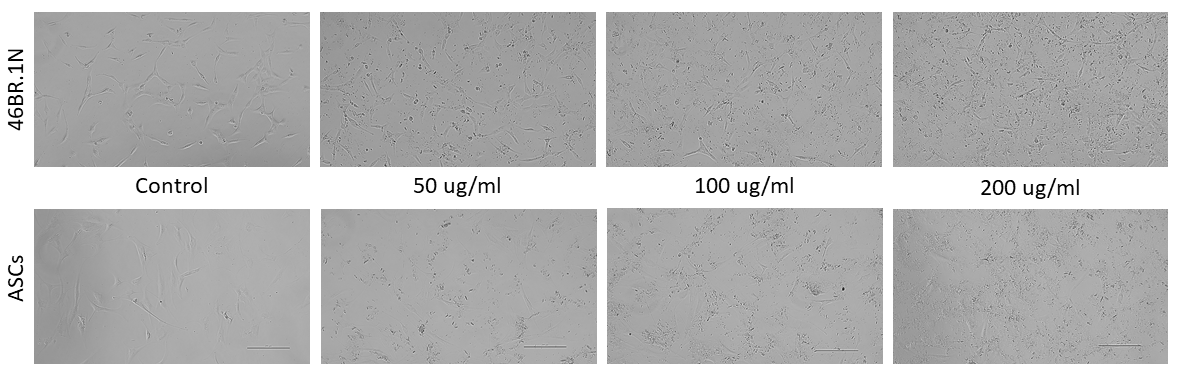


**Figure 2S.** Morphology of fibroblast 46BR.1N and ASCs after stimulation by MCC (24 h).


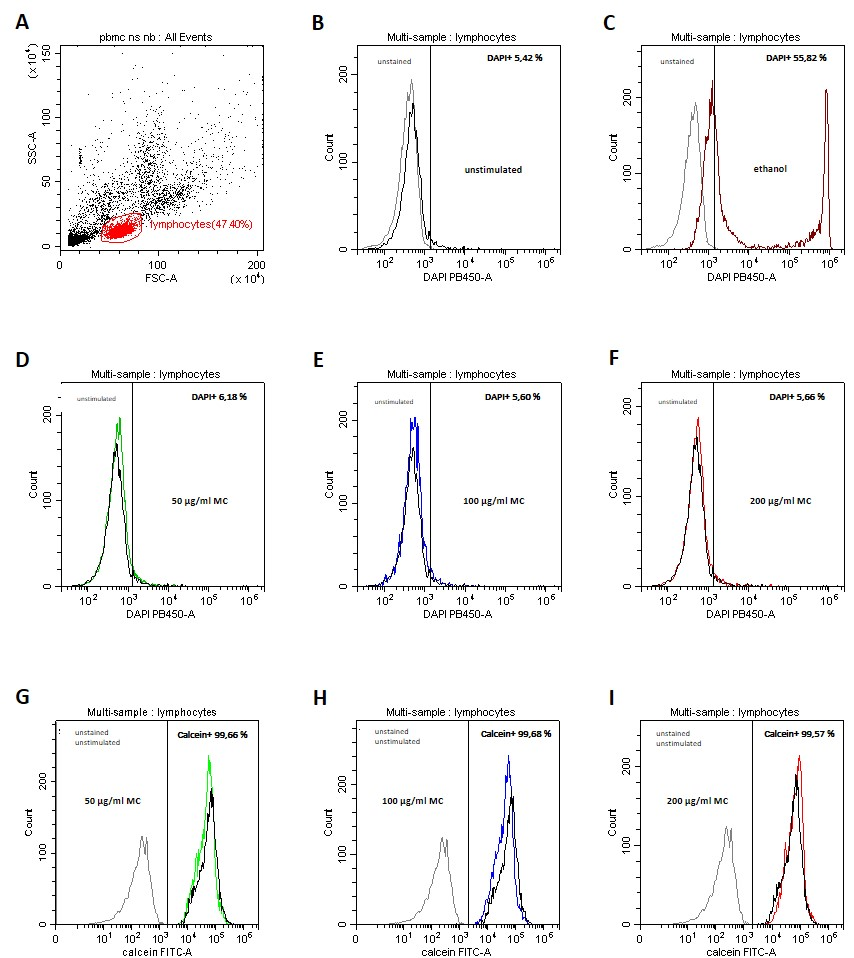


**Figure 3S.** Effect of the MCC on PBMC viability. This was checked by DAPI ( DAPI+ dead cells; B-F) and Calcein AM (alive cells; G-I) staining. Representative dot plot of PBMC with gated lymphocytes (A). Representative overlay histograms analysis of unstained and unstimulated cells (B), and DAPI-stained: ethanol-treated (C) as a positive control with dead cells. On histograms D-F, cells were stimulated by MCC in different concentrations (50 µg/ml – D (green line), 100 µg/ml – E (blue line), 200 µg/ml – F (red line)), and compared to the corresponding unstimulated sample. The histograms G-I show alive cells stained with Calcein AM stimulated by MCC (50 µg/ml – G (green line), 100 µg/ml – H (blue line), 200 µg/ml – I (red line)), compared with the unstained and unstimulated sample. Obtained values refer to the gated lymphocytes area.


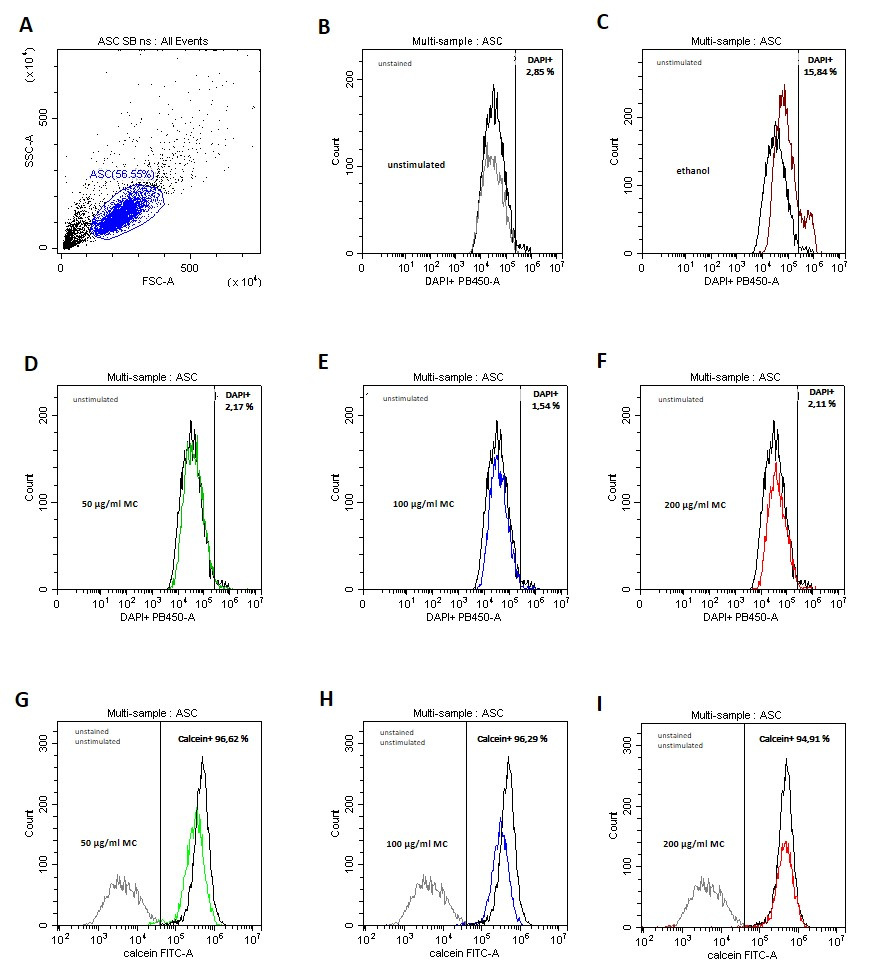


**Figure 4S.** Influence of the MCC on ASC viability. This was checked by DAPI (DAPI+ dead cells; B-F) and Calcein AM (Calcein+ alive cells; G-I) staining. Representative dot plot of gated ASCs (A). Representative overlay histograms analysis of unstained cells (B), and DAPI-stained: ethanol-treated (C) as a positive control with dead cells. On histograms D-F, cells were stimulated by MCC in different concentrations (50 µg/ml – D (green line), 100 µg/ml – E (blue line), 200 µg/ml – F (red line)), and compared to the corresponding unstimulated sample. The histograms G-I show alive cells stained with Calcein AM –stimulated by MCC (50 µg/ml – G (green line), 100 µg/ml – H (blue line), 200 µg/ml – I (red line)), compared with the unstained and unstimulated samples. Obtained values refer to the gated ASCs area.


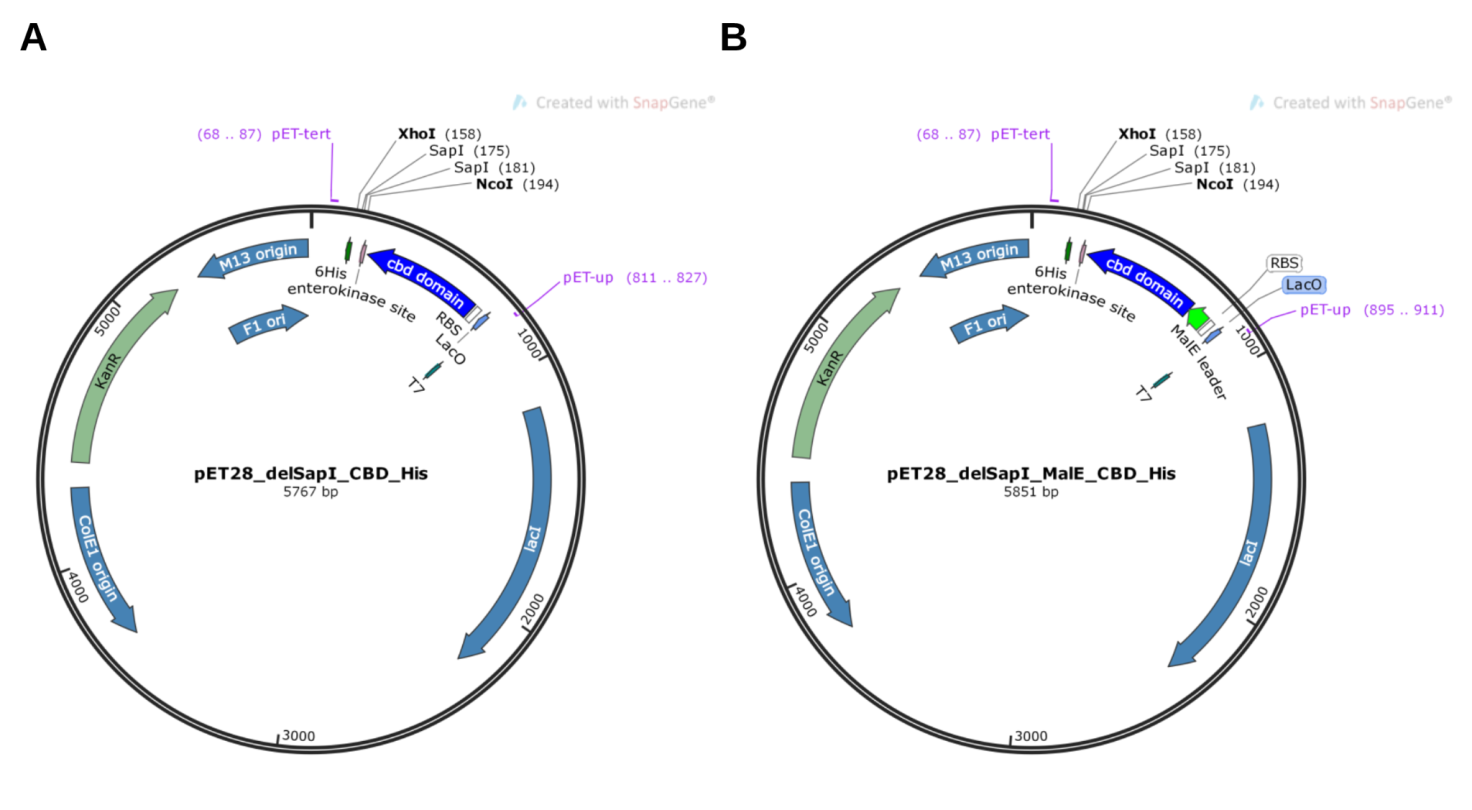


**Figure 5S.** New plasmid vector maps. Panel A. pET28_delSapI_CBD_His**.** Panel B. pET28_delSapI_MalE_CBD_His.


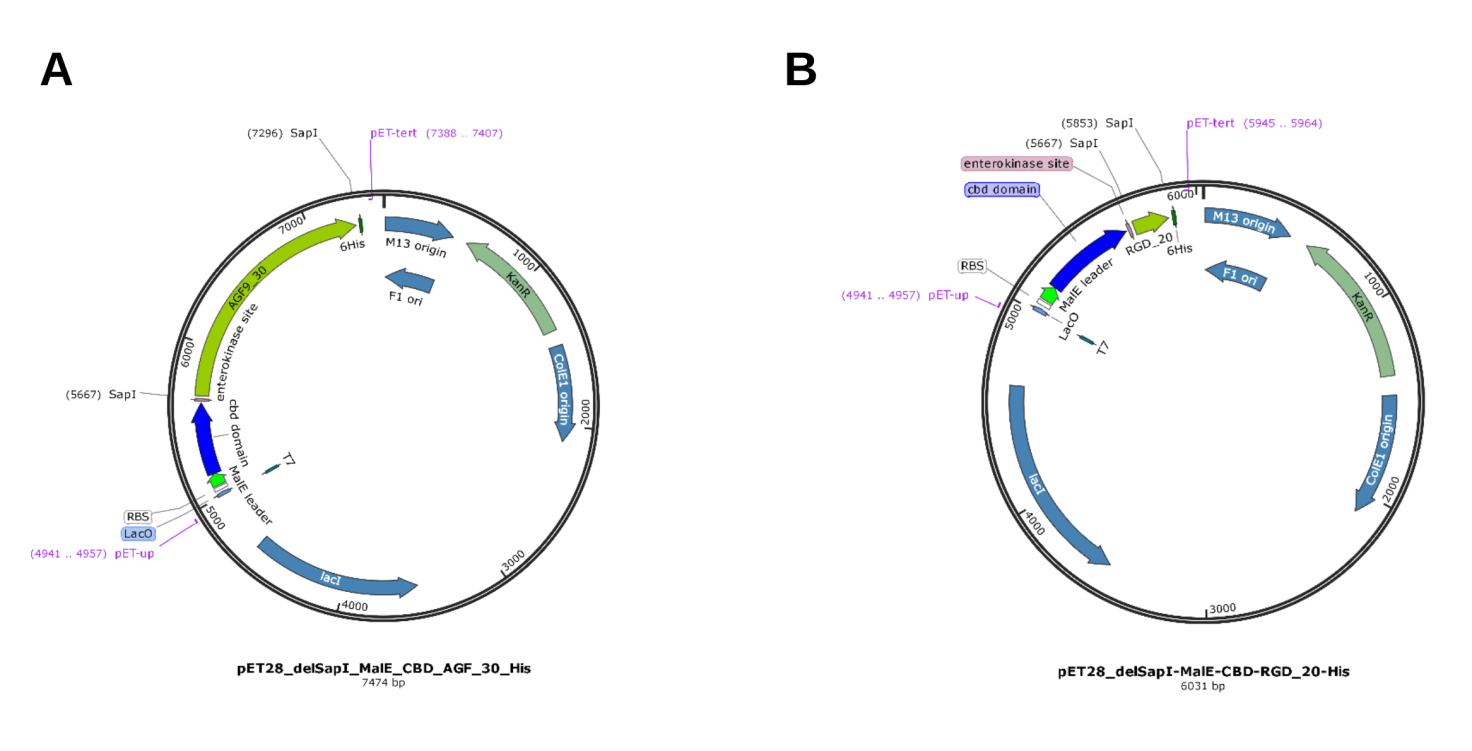


**Figure 6S.** New plasmid maps with AGF_poliepitopic and RGD_poliepitopic proteins. Panel A. Map of pET28_delSapI_MalE_CBD_AGF_30_His**.** Panel B. Map of pET28_delSapI_MalE_CBD_RGD_20_His.
